# Macrophage functional plasticity in periodontitis: microenvironmental regulation, disease progression, and therapeutic targeting

**DOI:** 10.3389/fimmu.2026.1973755

**Published:** 2026-10-12

**Authors:** Ziqi Gu, Xinyu Li, Gulinur Awuti

**Affiliations:** 1 Department of Periodontology, The First Affiliated Hospital of Xinjiang Medical University (Affiliated Stomatological Hospital), Urumqi, Xinjiang, China; 2 Chengdu University of Traditional Chinese Medicine, Chengdu, China; 3 School of Medical and Life Sciences, Chengdu University of Traditional Chinese Medicine, Chengdu, China

**Keywords:** functional plasticity, immune microenvironment, macrophages, periodontal regeneration, periodontitis, resolution of inflammation

## Abstract

Periodontitis is a chronic inflammatory disease driven by periodontal microbial dysbiosis and dysregulated host immune responses. Its progression is characterized by persistent inflammation, destruction of periodontal soft tissues, disrupted alveolar bone remodeling, and impaired tissue repair. As key effector cells within the periodontal immune microenvironment, macrophages contribute to pathogen clearance, inflammatory regulation, bone homeostasis, and tissue repair. Macrophage responses are highly heterogeneous and context dependent, extending well beyond the conventional M1/M2 dichotomy. This review focuses on macrophage functional plasticity in periodontitis, defined here as context-dependent changes in macrophage functional responses rather than heterogeneity or state-associated signatures alone, and examines how periodontal microorganisms and their virulence factors, host inflammatory signals, hypoxia and oxidative stress, immunometabolic changes, and epigenetic and post-transcriptional mechanisms regulate these responses. We further discuss how these responses contribute to inflammatory amplification, extracellular matrix degradation, alveolar bone resorption, resolution of inflammation, and periodontal tissue repair. Emerging evidence indicates that macrophage dysregulation in periodontitis extends beyond an imbalance between pro-inflammatory and repair-associated functions to include alterations in macrophage subset composition and selected functional programs. Direct evidence tracking transitions within macrophage populations in periodontal lesions, however, remains limited. The strategies reviewed here target inflammatory pathways, immunometabolism, intercellular communication, and the local tissue microenvironment. Linking changes in macrophage function to tissue outcomes may clarify the immunopathogenesis of periodontitis and inform treatments that promote inflammation resolution and periodontal regeneration.

## 1 Introduction

Periodontitis is a chronic, multifactorial inflammatory disease associated with dysbiosis of the dental plaque biofilm and characterized by the progressive destruction of the tooth-supporting tissues (1). Periodontitis is highly prevalent worldwide. In 2021, an estimated 1.07 billion people were affected by severe periodontitis, nearly twice the number reported in 1990. Although the age-standardized prevalence remained broadly stable over the same period, the absolute number of affected individuals and the total burden of disability-adjusted life years (DALYs) continued to increase (2). The onset and progression of periodontitis are not simply attributable to dental plaque accumulation but rather arise from the sustained interplay between periodontal microbial dysbiosis and dysregulated host immune-inflammatory responses (3).

Macrophages are key components of the innate immune system. In addition to clearing pathogens, dead cells, and cellular debris, they regulate inflammatory responses, tissue homeostasis, and tissue repair through the secretion of cytokines, growth factors, and other signaling molecules (4, 5). Macrophage phenotypes and functions are not fixed but are dynamically shaped by their cellular origin, tissue niche, and local environmental cues (4). Traditionally, macrophages have been broadly categorized into pro-inflammatory M1-like and repair-associated M2-like states. Although this framework is useful for conceptualizing their distinct functional tendencies, macrophages *in vivo* typically occupy dynamic, continuous, and overlapping functional states that cannot be readily divided into two mutually exclusive categories (6).

Under persistent microbial and inflammatory stimulation in periodontitis, local immune signals and the tissue microenvironment dynamically shape macrophage functional states, thereby influencing local inflammation and alveolar bone resorption (7). Persistent M1-like pro-inflammatory responses are closely associated with amplified inflammatory responses and periodontal tissue damage (8). In contrast, M2-like repair-associated responses may contribute to the regulation and resolution of inflammation, alveolar bone repair, and periodontal tissue regeneration (9). These stimulus-defined tendencies describe selected aspects of inflammation and repair, but do not establish a universal M1/M2 balance that determines tissue outcome. This review examines how the periodontal microenvironment regulates macrophage function, how these responses contribute to tissue damage and repair, and how they may be therapeutically targeted. In this context, macrophage functional plasticity refers to context-dependent changes in macrophage functional responses rather than to heterogeneity or state-associated signatures alone. Accordingly, we distinguish descriptive evidence of macrophage states from experimentally demonstrated functional changes and from evidence supporting transitions between functional states or functional reprogramming.

## 2 Biological characteristics and functional heterogeneity of macrophages

### 2.1 Origins and fundamental functions of macrophages

Macrophages are key myeloid cells in periodontal tissues that contribute to local tissue homeostasis and host responses to microbial challenges (7). Studies of oral tissues generally indicate that periodontal macrophages arise from two major sources: tissue-resident macrophages established during embryonic development and macrophages differentiated from circulating monocytes (10). Tissue-resident macrophages colonize tissues during development and can persist long term, with their populations maintained in part through local self-renewal (11). In contrast, circulating monocytes can infiltrate local tissues during inflammation or tissue injury and differentiate into macrophages, thereby replenishing the local macrophage population (12). Despite their distinct developmental origins, macrophages share core phagocytic and immunoregulatory functions. They clear microorganisms, apoptotic cells, and tissue debris, while contributing to tissue homeostasis and repair through the release of growth factors and other signaling molecules (4). In experimental periodontitis, both monocytes and macrophages accumulate in gingival tissues, suggesting that inflammation-driven recruitment may contribute to the expansion of the local macrophage population (13). Single-cell transcriptomic analyses of human gingival tissues further reveal pronounced transcriptional and signaling heterogeneity among macrophages associated with periodontitis (7). Collectively, macrophages in periodontal tissues comprise both tissue-resident and inflammation-recruited populations, whose relative composition and state-associated profiles may vary with the local microenvironment and disease progression. Human transcriptomic observations describe cellular composition and state, whereas developmental origin and recruitment require evidence from lineage or trafficking studies; these forms of evidence should not be treated as interchangeable.

### 2.2 Macrophage polarization and functional heterogeneity

In periodontitis, macrophages contribute not only to host defense against infection but also to the resolution of inflammation and tissue repair. Their functional states have traditionally been conceptualized within the M1/M2 framework. Microbial components can induce M1-associated responses, as can host-derived cytokines such as IFN-γ, thereby promoting the production and release of pro-inflammatory mediators (14). This functional state enhances pathogen clearance, antigen presentation, and Th1-type immune responses (15). However, when M1-like responses remain predominant, the sustained release of pro-inflammatory mediators and matrix-degrading enzymes can amplify local inflammation and promote periodontal tissue destruction (8). In contrast, cytokines such as IL-4 and IL-13 can promote M2-associated responses associated with the regulation and resolution of inflammation, extracellular matrix remodeling, and tissue repair (16). A study using these operational categories reported a lower M2/M1 marker ratio in periodontitis-affected human gingiva and an association between a lower Arg-1/iNOS ratio and greater bone loss in a mouse model (9). These observations link selected M1/M2-associated markers to periodontal destruction within the study’s classification scheme. They do not, however, show that all periodontal macrophages fall into two distinct groups or establish the functions of individual cells.

The M1/M2 framework describes responses to selected experimental stimuli but does not provide a comprehensive taxonomy of macrophages in periodontal tissues. In the human M-CSF-derived macrophage culture system examined by Jaguin and colleagues, CD206 did not discriminate between the tested M1 and M2 conditions (16), and human monocyte-derived macrophages show stimulus-dependent transcriptional programs (14). Mouse bone-marrow-derived macrophages exposed to mixed signals can co-express markers assigned to opposing categories (17). In a mouse periodontal infection model, NOS2- and CD206-associated features can increase together despite an overall inflammatory response (18). Human gingival single-cell analyses likewise resolve heterogeneous transcriptional states that are not adequately represented by mutually exclusive M1 and M2 labels (7). These findings indicate that periodontal macrophages should be described in relation to their local environment and functional state, rather than solely by M1/M2 labels. Cell abundance, marker expression, and transcriptional profiles can help characterize these states, but cannot replace direct measurements of function. Accordingly, changes in these state-associated features should not, by themselves, be interpreted as evidence that individual macrophages have undergone a functional state transition.

Chen and colleagues identified periodontitis-enriched macrophage clusters with distinct metabolic and regulatory signatures; enrichment of both glycolytic and oxidative-phosphorylation programs argues against assigning each cluster to a single canonical metabolic phenotype (19). Their cell-communication and pseudotime analyses nominate interactions and trajectories, but do not directly establish ligand activity or lineage conversion *in vivo*. Validation of selected genes in inflammatory macrophage cultures supports their responsiveness to stimulation, rather than proving that the inferred tissue states are stable, functionally distinct lineages. The authors acknowledged possible sample overlap or non-independence between GSE10334 and GSE16134, while also reporting validation in additional cohorts, including GSE106090 and GSE173078 (19).

A complementary study compared periodontal health, disease, and resolution after removal of the inflammatory stimulus in mice. It identified an immunoregulatory macrophage subgroup enriched for Cd74 that expanded during resolution; flow cytometry and tissue staining supported the presence of this population. Reanalysis of a human periodontal dataset identified a corresponding post-treatment-associated population enriched for CD74 (20). Thus, CD74-associated states should be interpreted in their temporal and tissue context, rather than classified automatically as disease-promoting macrophages. Importantly, this study did not establish that CD74 itself is necessary or sufficient for periodontal resolution: targeted perturbation and functional assays remain required (20). The expansion of a CD74-enriched population during resolution may reflect changes in recruitment, survival, proliferation, or macrophage state, and the available data do not distinguish among these possibilities.

Together, these datasets support transcriptional heterogeneity involving inflammatory signaling, antigen processing, and metabolism. Such profiles do not directly establish efferocytosis or tissue-supportive function. The human single-cell analyses in the Chen study and the human reanalysis in the CD74 study both used GSE171213, so these components are not independent human-cohort replications (19, 20). Cluster labels should therefore be reported with their species, sampling condition, and analytical definition. For therapeutic studies, a shift in marker expression should be distinguished from improved macrophage function and from a measurable benefit to periodontal tissues.

## 3 Regulation of macrophage functional states by the periodontal microenvironment

### 3.1 Periodontal microorganisms and their virulence factors

Cell wall components, adhesive structures, and outer membrane vesicles (OMVs) derived from periodontal microorganisms can modulate macrophage responses. In macrophages exposed to defined prior stimulation, Porphyromonas gingivalis lipopolysaccharide (LPS) elicits stimulus-dependent changes in inflammatory mediator and chemokine production, whereas canonical M1- or M2-associated markers do not change uniformly (21). These findings support context-dependent functional responsiveness to a subsequent microbial stimulus, but do not by themselves establish conversion between discrete macrophage states. Fimbria-mediated receptor interactions primarily influence the intracellular fate of bacteria following uptake. Native fimbriae activate complement receptor 3 (CR3) through TLR2-dependent signaling, thereby promoting the internalization of *Porphyromonas gingivalis* by macrophages while suppressing IL-12p70 production and prolonging bacterial intracellular persistence (22). OMVs can deliver concentrated cargos, including LPS, proteases, and lipids, to host cells. *In vitro* comparisons have shown that OMVs induce more robust inflammatory mediator release and inflammasome activation than intact bacterial cells (23). The composition of OMV cargo further shapes their immunomodulatory effects. Gingipains can selectively alter the secretion profiles of inflammatory mediators and chemokines, whereas sphingolipids may limit OMV uptake and attenuate early recognition mediated by the TLR2–MyD88 signaling pathway (24, 25). Collectively, these mechanisms differentially regulate inflammatory mediator production, pathogen internalization, inflammasome activation, and intracellular clearance, demonstrating that macrophage responses to Porphyromonas gingivalis vary according to the microbial signal and experimental context.

Similar multilayered regulation is also observed with other periodontitis-associated bacteria. Outer membrane vesicles (OMVs) derived from Treponema denticola and Tannerella forsythia can be internalized by macrophages and activate NF-κB signaling and inflammasome-associated responses (26). In mouse bone-marrow-derived macrophages, Fusobacterium nucleatum OMVs increased TNF-α and iNOS expression, NF-κB p65 phosphorylation, and ROS production. The original study described this response as M1-like polarization (27). Here, the term refers to the pro-inflammatory response measured under those experimental conditions; it does not identify a stable macrophage subset in human periodontal tissue. Beyond vesicle-mediated delivery, peptidoglycan from Treponema denticola can promote the production of inflammatory mediators, chemokines, and MMP-9, thereby simultaneously enhancing the inflammatory secretory and proteolytic activities of macrophages (28). In contrast, the effects of Aggregatibacter actinomycetemcomitans on macrophages are mediated largely by leukotoxin (Ltx). This toxin induces pro-inflammatory cell death through P2X7- and caspase-1-dependent mechanisms and promotes the release of bioactive IL-1β and IL-18 (29). Comparisons among strains with different levels of leukotoxin production further support Ltx as a major determinant of A. actinomycetemcomitans-induced IL-1β release from macrophages (30).

### 3.2 Host-derived inflammatory and immunoregulatory signals

Following the initiation of local immune responses by microbial signals, host-derived mediators released by gingival tissue cells and infiltrating immune cells further modulate macrophage responses. CCL2 is a key monocyte chemoattractant that promotes the recruitment of circulating monocytes to periodontal tissues through CCR2-dependent signaling (13). In diabetes-associated periodontitis, persistently elevated CCL2 levels are accompanied by sustained accumulation of pro-inflammatory Ly6C^hi^ monocytes in periodontal tissues (31). Following their recruitment to periodontal lesions, monocytes are further modulated by local complement signaling. C3a, generated through C3 cleavage, can bind to C3aR on macrophages, activate NF-κB signaling, and enhance CD86-associated pro-inflammatory features (5). Under *P. gingivalis* stimulation, crosstalk between C5aR and TLR2 signaling can suppress IL-12p70-mediated antimicrobial responses while enhancing the production of IL-1β, IL-6, and TNF-α (32). This mechanism may facilitate bacterial persistence and sustain local inflammation.

Beyond complement signaling, local cytokines and acute-phase inflammatory proteins can further sustain macrophage inflammatory responses. B-cell activating factor (BAFF), a member of the TNF superfamily expressed by monocytes, macrophages, stromal cells, and other cell types, contributes to this process. BAFF blockade or knockdown reduces TNF-α and iNOS expression in macrophages following stimulation with P. gingivalis LPS, supporting a role for BAFF in sustaining pro-inflammatory responses under these experimental conditions. These findings do not, however, demonstrate a transition between macrophage functional states (33). Fibroblast-derived serum amyloid A1 (SAA1) exerts both chemotactic and pro-inflammatory effects, promoting macrophage migration and enhancing NF-κB activity and the expression of TNF-α and IL-6, thereby reinforcing inflammatory crosstalk between stromal cells and macrophages (34). Regulatory signals that restrain excessive inflammation are also present within periodontal lesions. Type I interferons (IFN-I) produced by gingival fibroblasts and keratinocytes can promote CD169^+^MERTK^+^-associated macrophage features through IFNAR1–IRF7 signaling and increase the expression of CCL18 and IL-10 (35). These findings support an immunoregulatory response profile under the tested conditions rather than demonstrating a complete functional state transition (35).

### 3.3 Hypoxia, oxidative stress, and immunometabolic reprogramming

Cheng and colleagues detected increased HIF-1α staining in human periodontitis specimens and hypoxyprobe localization in an LPS-induced mouse periodontal inflammation model (36). These findings support the presence of a hypoxia-associated tissue environment, but do not establish macrophage-specific HIF-1α causality; much of the mechanistic work in that study concerned gingival fibroblasts. HIF-1α staining also cannot be translated directly into an exact local oxygen concentration. Hypoxia, inflammatory signaling, and altered tissue perfusion should therefore be considered together when interpreting the periodontal HIF-1α axis (36, 37).

In the murine J774A.1 macrophage cell line, exposure to 2% O_2_ was associated with HIF-1α activation, increased glycolysis and ROS production, and impaired migration while bacterial uptake and intracellular control were retained (38). This defined culture condition isolates an oxygen-dependent response; it does not reproduce the spatial gradients, changing perfusion, multispecies biofilms, or interacting cell populations within a periodontal lesion. HIF-1α-related interventions also yield context-dependent results. Suppression of HIF-1α-associated PI3K/Akt signaling and glycolysis attenuated inflammatory responses in the phloretin study (39), whereas the prolyl-hydroxylase inhibitor and HIF-1α stabilizer DMOG reduced inflammatory macrophage features and alveolar bone loss in a murine periodontitis model (40). These interventions differ in their targets and experimental conditions, so their findings do not establish a universal benefit from either increasing or decreasing HIF-1α. Cell-specific perturbation, oxygen mapping, and temporal analysis are needed to determine when this pathway sustains injury or alters macrophage functional responses.

In addition to hypoxia-induced ROS production, impaired mitochondrial quality control can further exacerbate intracellular oxidative stress. In macrophage assays, stimulation with P. gingivalis LPS and IFN-γ reduces TUFM expression and mitophagic activity, accompanied by mitochondrial dysfunction and ROS accumulation. Conversely, restoration of TUFM-mediated mitophagy improves mitochondrial function and attenuates pro-inflammatory macrophage features (41). These cellular disturbances are not confined to the mitochondria. Following stimulation with P. gingivalis LPS, increased UCHL1 expression is accompanied by loss of mitochondrial membrane potential, reduced ATP production, and endoplasmic reticulum stress. Conversely, inhibition of UCHL1 alleviates mitochondrial dysfunction, ROS accumulation, and endoplasmic reticulum stress (42). Impaired mitochondrial clearance and organelle stress may act in concert to sustain a high oxidative burden and prolong inflammatory responses in macrophages.

Mitochondrial dysfunction can also alter glucose utilization and the availability of tricarboxylic acid (TCA) cycle intermediates. In cell-based metabolic assays, P. gingivalis and its OMVs can enhance glycolysis, lactate production, and mitochondrial reactive oxygen species (mtROS) generation, while reducing mitochondrial oxygen consumption, spare respiratory capacity, and the expression of TCA cycle-related genes (23). Apabetalone-mediated inhibition of glucose uptake and the expression of HK2, GLUT1, and PFKFB3 restores TCA cycle activity and mitochondrial respiration, accompanied by attenuation of M1-like inflammatory features (43). Enhanced glycolysis is therefore not an isolated metabolic alteration but occurs in parallel with impaired mitochondrial metabolism and persistent inflammatory responses. At the same time, remodeling of the TCA cycle alters the balance of metabolites with immunoregulatory properties. P. gingivalis reduces the α-ketoglutarate (α-KG)/succinate ratio by suppressing α-KG production and attenuates IL-4-induced regulatory activation, whereas α-KG supplementation can partially restore this response (44). In general macrophage models, α-KG has also been implicated in Jmjd3-dependent epigenetic reprogramming; however, direct evidence for this mechanism in periodontitis remains limited (45).

### 3.4 Epigenetic and post-transcriptional regulation

Metabolic disturbances within the periodontal microenvironment can induce relatively persistent epigenetic changes. In diabetes-associated periodontitis, hyperglycemia suppresses dihydrofolate reductase (DHFR)-dependent one-carbon metabolism, thereby reducing the availability of S-adenosylmethionine (SAM) and decreasing DNA methylation associated with NF-κB p65. These epigenetic alterations may sustain the inflammatory macrophage phenotype even after the metabolic stimulus has subsided (46). This persistence supports metabolic memory under the tested conditions, but does not by itself establish a general loss of macrophage functional plasticity or a defined transition between functional states. In THP-1-derived macrophages stimulated with *P. gingivalis* LPS and IFN-γ, TET1 expression is reduced; nevertheless, further TET1 knockdown suppresses NF-κB activation and decreases the expression of IL-6, TNF-α, and CCL2 (47). In addition to DNA methylation, histone acetylation can modulate the transcriptional activity of inflammation-related genes. Lysine acetyltransferase 6A (KAT6A) maintains histone H3 lysine 27 acetylation (H3K27ac) at NF-κB-binding regions within the IL-1β and TNF-α promoters, thereby promoting inflammatory transcription in response to *P. gingivalis* LPS (48). This regulatory mechanism also extends to the biogenesis of non-coding RNAs. Hyperglycemia increases histone H3 lysine 56 acetylation (H3K56ac) by downregulating sirtuin 6 (SIRT6), thereby promoting the transcription and maturation of the miR-216/217 cluster. The resulting suppression of DEL-1 and CD36 impairs macrophage-mediated clearance of apoptotic cells (49). Because apoptotic-cell clearance was directly assessed, these findings provide functional evidence beyond changes in macrophage-associated markers.

Following transcription, the expression of inflammation-related genes remains subject to post-transcriptional regulation through RNA stability and miRNA-mediated mechanisms. N^6^-methyladenosine (m^6^A) modification contributes to this regulation by influencing mRNA fate. The m^6^A reader protein IGF2BP2 limits inflammatory mediator release during the early stages of periodontitis, whereas at later stages it modulates myeloid cell function by stabilizing Cd5l and Cd36 mRNAs (50). However, whether this effect directly depends on m^6^A recognition remains unclear. In another study, the m^6^A methyltransferase METTL3 reduced the stability of TNFAIP3 mRNA, which encodes a negative regulator of inflammation, thereby decreasing NEK7 ubiquitination and promoting assembly of the NLRP3 inflammasome (51). This mechanism was established primarily in gingival fibroblasts and subsequently validated in immortalized bone marrow-derived macrophages. Beyond the regulation of mRNA stability, microRNAs (miRNAs) modulate specific signaling pathways by recognizing target sequences within the 3′ untranslated region (3′UTR). EV-associated miR-143-3p derived from inflammatory periodontal ligament stem cells (PDLSCs) targets phosphoinositide 3-kinase γ (PI3Kγ), suppressing PI3K/AKT signaling while enhancing NF-κB activity and thereby promoting M1-like inflammatory features (52). This repression depends on the retention of the corresponding miRNA-binding site within the target mRNA. P. gingivalis induces alternative polyadenylation (APA), generating Selenok mRNA isoforms with a shorter 3′UTR that lack the miR-320-3p-binding site, thereby relieving miR-320-3p-mediated suppression of Selenok expression (8).

Periodontal microorganisms, host-derived inflammatory mediators, and local metabolic stress interact to shape the antimicrobial, pro-inflammatory, and reparative functions of macrophages through integrated regulation of receptor signaling, metabolic reprogramming, chromatin states, and RNA-mediated processes. Human tissue expression and spatial associations establish disease relevance, whereas cell-based perturbations and animal experiments provide different tests of mechanism and tissue consequences. Neither observational associations nor inferred cell communication alone establishes a causal pathway in patients. Likewise, changes in signaling, metabolism, chromatin state, or RNA regulation identify mechanisms that may alter macrophage responses, but do not by themselves demonstrate a transition between functional states. However, most current studies rely on single-stimulus models and static endpoints, limiting their ability to capture the temporal dynamics of these mechanisms across different stages of disease and macrophage subsets. Future studies should therefore validate key regulatory mechanisms in models that preserve cellular heterogeneity and disease progression, while distinguishing the pathways that predominantly contribute to the initiation and persistence of inflammation from those responsible for impaired inflammatory resolution (**Figure 1**).

**Figure 1:**
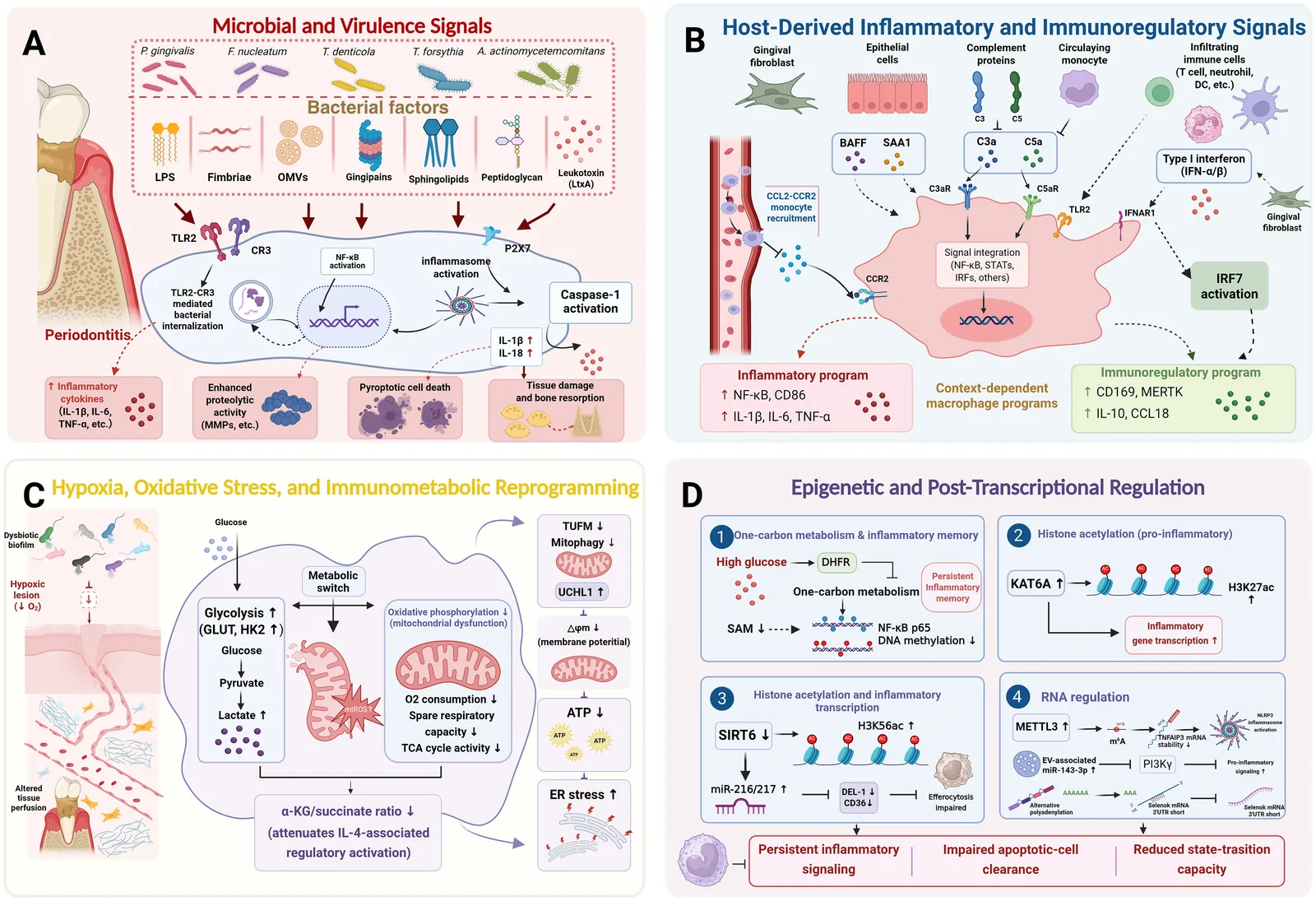
Microenvironmental regulation of macrophage functional plasticity in periodontitis. Macrophage responses in periodontitis are shaped by interconnected microbial, inflammatory, metabolic, and molecular signals within the periodontal microenvironment. **(A)** Periodontal microorganisms and their virulence factors, including LPS, fimbriae, outer membrane vesicles (OMVs), gingipains, sphingolipids, peptidoglycan, and leukotoxin, activate or subvert pattern-recognition and intracellular signaling pathways in macrophages, thereby influencing inflammatory mediator production, pathogen internalization and clearance, proteolytic activity, inflammasome activation, and inflammatory cell death. These responses contribute to persistent inflammation, tissue damage, and alveolar bone resorption. **(B)** Host-derived signals further modulate macrophage recruitment and response-associated features. CCL2–CCR2 signaling promotes monocyte recruitment, whereas complement components, BAFF, SAA1, and other inflammatory mediators amplify macrophage inflammatory responses. In parallel, type I interferon signals associated with gingival fibroblasts and keratinocytes can promote immunoregulatory features, illustrating the context-dependent coexistence of pro-inflammatory and inflammation-regulating macrophage responses. **(C)** Hypoxia, oxidative stress, and metabolic reprogramming reshape macrophage bioenergetics. Their functional consequences depend on oxygen exposure, accompanying stimuli, and the experimental model. Increased glycolysis, mitochondrial ROS accumulation, impaired oxidative phosphorylation, disrupted mitochondrial quality control, reduced ATP production, endoplasmic reticulum stress, and alterations in TCA-cycle metabolites collectively favor persistent inflammatory dysfunction and may impair repair-associated responses. **(D)** Epigenetic and post-transcriptional mechanisms regulate the persistence and responsiveness of macrophage-associated programs. Altered one-carbon metabolism and DNA methylation, histone acetylation, miRNA-dependent regulation, m^6^A-associated RNA regulation, and 3′UTR remodeling influence inflammatory gene expression and selected macrophage functions, including efferocytosis, in the cited models. The relationships illustrated in this panel summarize regulatory associations reported across different experimental systems and should not be interpreted as direct evidence of state transitions within individual macrophages. M1-like and M2-like labels are used as operational shorthand for selected response-associated features and do not represent mutually exclusive identities or demonstrated state conversion.

## 4 Roles of macrophage functional states in the pathogenesis and progression of periodontitis

### 4.1 Inflammatory responses and periodontal soft-tissue damage

Pro-inflammatory macrophage-associated features have been linked to periodontitis severity, although these associations cannot be reduced to a fixed M1/M2 ratio. In human gingival tissues, the ratio of M1- to M2-associated markers correlates positively with IL-1β and MMP-9 levels and probing depth (53). These measurements associate macrophage-related signatures with inflammatory burden and matrix-degrading activity, but do not directly demonstrate a change in macrophage function. Single-cell colocalization analyses have also identified macrophages co-expressing iNOS and CD206, without consistent differences in the proportions of M1- or M2-like cells between periodontitis and healthy tissues (54). Such co-expression indicates overlapping phenotypic features rather than direct evidence that individual macrophages are undergoing a functional state transition. In animal models, reductions in M1-like macrophage abundance are accompanied by decreased macrophage infiltration and IL-1β expression within periodontal lesions (55). Reduced pro-inflammatory macrophage-associated responses are also accompanied by improved epithelial integrity, reduced neutrophil infiltration, and decreased collagen degradation (5). These findings should be interpreted using inflammatory readouts and tissue outcomes together, rather than a single polarization marker.

In the setting of persistent inflammation, activated macrophages can further damage gingival structural cells and disrupt extracellular matrix homeostasis. In a mouse-cell co-culture model, macrophages stimulated with Fusobacterium nucleatum OMVs increased gingival fibroblast apoptosis and membrane damage (27). Subsequently, macrophage-derived inflammatory mediators can stimulate fibroblasts to produce matrix metalloproteinases while suppressing the expression of type I and III collagens, thereby driving the progression from structural cell injury to extracellular matrix loss (56). In human gingival tissues, increased numbers of CD68^+^ cells and elevated levels of multiple MMPs are closely associated with reduced collagen area, providing clinicopathological support for this mechanistic link (57). Together, the cell experiments support mechanisms of structural injury, while the human tissue observations show association rather than intervention-based causality. Pro-inflammatory macrophages may sustain inflammatory cell infiltration, damage structural cells, and enhance extracellular matrix degradation, thereby transforming a local antimicrobial response into persistent periodontal soft-tissue destruction.

### 4.2 Alveolar bone resorption and dysregulated bone remodeling

Alongside ongoing soft-tissue damage, macrophage-mediated effects extend to the regulation of alveolar bone remodeling. In the cited mouse infection model, Porphyromonas gingivalis infection is associated with an increased abundance of CD86^+^ macrophages within periodontal lesions. Macrophage depletion reduces both bacterial burden and alveolar bone resorption, suggesting that macrophages serve as an important link between persistent infection, sustained inflammation, and pathological bone loss (58). Consistent with these findings, local enrichment of macrophages with M2-associated features is accompanied by reduced RANKL expression, fewer osteoclasts, and attenuated alveolar bone resorption across different models of periodontitis (59).

Dysregulated bone remodeling is also reflected by the failure of bone regeneration to recover promptly after resorption. Although macrophage depletion limits bone loss during the inflammatory phase, it also impairs bone regeneration after removal of the inflammatory stimulus. In contrast, interventions associated with increased M2-like macrophage features are accompanied by reduced osteoclastic activity and enhanced new bone formation (60). These population-level changes and tissue outcomes support an association between macrophage phenotype and bone remodeling, but do not by themselves demonstrate a functional state transition within existing macrophages. These bidirectional effects may be mediated through crosstalk between macrophages and bone-associated cells. EVs derived from M2-like macrophages deliver IL-10 mRNA, thereby promoting osteogenic differentiation of bone marrow stromal cells while inhibiting the differentiation of bone marrow-derived macrophages into osteoclasts. Both effects are markedly attenuated following blockade of the IL-10 receptor, supporting a central role for IL-10 signaling in this intercellular communication (61). Beyond the conventional M1/M2 framework, specific repair-associated macrophage subsets also contribute to the regulation of bone homeostasis. Tim4 deficiency reduces the abundance of CD301b^+^ macrophages and exacerbates alveolar bone resorption, further indicating that the M1/M2 paradigm alone is insufficient to fully capture the diverse roles of macrophages in bone remodeling (6). Alveolar bone loss in periodontitis is driven by both enhanced osteoclastic activity and insufficient osteogenic support.

### 4.3 Resolution of inflammation and periodontal tissue repair

Even after bone resorption is controlled during the inflammatory phase, subsequent bone formation and periodontal tissue reconstruction following removal of the inflammatory stimulus still depend on effective resolution of inflammation (60). Macrophage-mediated efferocytosis, the clearance of apoptotic cells, is a key component of this process. Following ligature removal, DEL-1-deficient mice continue to exhibit elevated levels of inflammatory mediators and alveolar bone loss. Further evidence from an acute inflammation model showed that DEL-1 enhances macrophage efferocytosis of apoptotic neutrophils (62). This directly measured increase in apoptotic-cell clearance supports a pro-resolving macrophage function, but does not by itself demonstrate a transition between macrophage functional states within periodontal lesions. Diabetes-associated periodontitis further highlights the link between impaired efferocytosis and defective tissue repair under metabolic stress. Excessive mitochondrial fission and accumulation of mitochondrial reactive oxygen species are associated with impaired macrophage efferocytosis. Pharmacological inhibition of mitochondrial fission partially restores efferocytic capacity and enhances the ability of macrophage-conditioned medium to support the migration and osteogenic differentiation of periodontal ligament stem cells (63). These experiments provide direct evidence of recovery in a defined macrophage function and its downstream tissue-supportive effects, although they do not establish coordinated reprogramming of the entire macrophage state. Collectively, these findings link impaired efferocytosis to delayed resolution of inflammation and reduced capacity to support tissue repair.

During periodontal healing, the distribution of macrophage-associated features changes as inflammation resolves. Following ligature removal, iNOS-associated pro-inflammatory features gradually decline, whereas Arg-1-associated reparative features increase, coinciding with reduced inflammatory mediator levels and improvements in alveolar bone architecture (64). These temporal changes are consistent with remodeling of the macrophage population during healing, but do not distinguish changes within existing macrophages from recruitment, survival, or loss of different macrophage populations. In periodontal ligament stem cell transplantation models, the early establishment of an anti-inflammatory microenvironment and an increased abundance of macrophages with repair-associated features are followed by the formation of new bone, cementum-like structures, and collagen fibers inserting into newly formed cementum (65). *In vitro* studies provide further evidence that macrophages regulate the functional behavior of periodontal structural cells. The secretory milieu generated by M2-like macrophages can enhance the fibrogenic and mineralization capacities of periodontal ligament cells (66). EVs isolated from RAW264.7 macrophages improved TNF-α-suppressed osteogenic and cementogenic differentiation of human periodontal ligament cells *in vitro*, with p38 MAPK implicated by inhibitor experiments; that study did not validate periodontal regeneration in animals (67). Through the clearance of apoptotic cells and regulation of periodontal structural cells, macrophages serve as a critical link between the resolution of inflammation and subsequent tissue repair (**Figure 2**).

**Figure 2:**
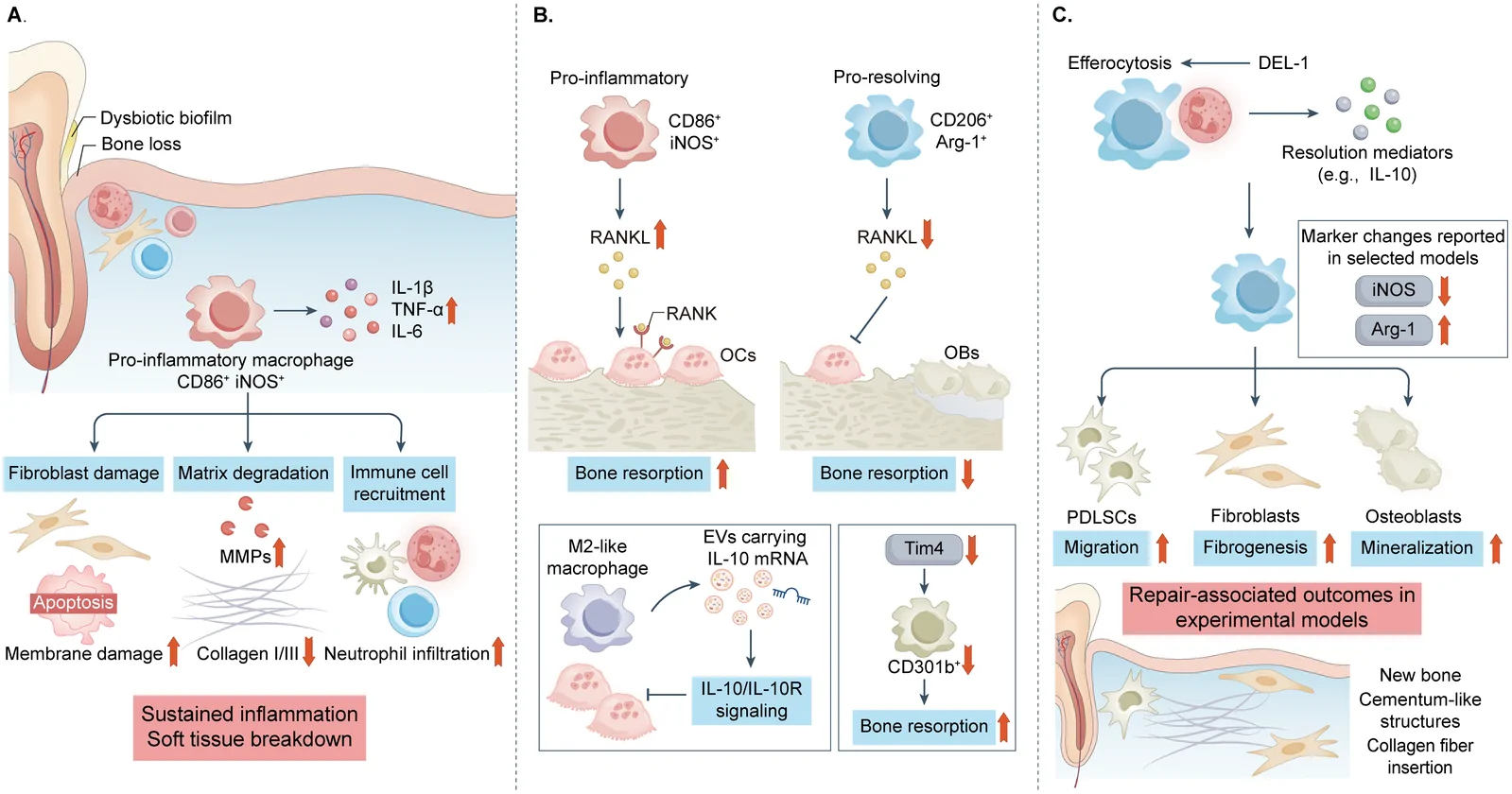
Macrophage-associated responses in periodontal inflammation, bone remodeling and tissue repair. **(A)** Macrophages with pro-inflammatory features, including CD86 and iNOS expression, are associated with increased production of IL-1β, TNF-α, and IL-6 in the illustrated experimental settings. These responses contribute to fibroblast injury, matrix degradation, and immune-cell recruitment, thereby promoting sustained inflammation and periodontal soft-tissue breakdown. **(B)** Macrophage-associated responses influence alveolar bone remodeling. Pro-inflammatory macrophage-associated responses have been linked to increased RANKL expression, osteoclastogenesis, and bone resorption, whereas repair-associated macrophage features have been associated with reduced RANKL expression and osteoclast activity in the cited models. M2-like macrophage-derived EVs containing IL-10 mRNA can promote osteogenic differentiation and suppress osteoclast differentiation through IL-10 receptor signaling. Loss of TIM4 is associated with reduced CD301b^+^ macrophages and increased bone resorption, highlighting the contribution of repair-associated macrophage populations to bone homeostasis. **(C)** DEL-1 supports macrophage efferocytosis of apoptotic neutrophils and pro-resolving responses. Repair-associated macrophage functions have been linked to PDLSC migration, fibroblast activity, and mineralization in distinct experimental settings. The marker changes and tissue outcomes summarized here do not establish a single universal causal sequence from efferocytosis to complete periodontal regeneration. The illustrated population features and functional effects were obtained across different experimental systems and do not indicate that individual macrophages sequentially pass through the depicted response states.

### 4.4 Systemic inflammatory context and smoking

Macrophage states in periodontal lesions should also be considered in the context of systemic inflammatory and metabolic conditions. Diabetes-associated studies link altered macrophage efferocytosis and metabolic regulation to impaired periodontal resolution (49, 63, 68). These findings do not establish a single macrophage program shared by every inflammatory comorbidity. COPD provides a further example of this contextual dependence: a study combining periodontal and pulmonary disease models implicated γδ T-cell-associated responses and pulmonary M2-marker-positive macrophages in aggravated lung pathology (69). Human bronchoalveolar observations supported clinical relevance but were subject to imbalanced exacerbation status, and the short-duration mouse model cannot establish the effect of periodontal treatment on human COPD. The pulmonary findings also caution against interpreting M2-associated markers as universally protective across tissues.

Smoking can influence both periodontal and pulmonary inflammation and must be considered as a shared exposure when interpreting their association. A human gingival spatial-transcriptomic study identified smoking-associated changes in macrophage transcriptional programs and endothelial proximity; accompanying endothelial CXCL12 perturbation in experimental systems provided additional mechanistic evidence (70). Nevertheless, human cross-sectional associations, inferred cell communication, and a nicotine-associated mouse model represent different evidentiary levels. They do not isolate a macrophage-mediated causal pathway from periodontitis to COPD. Future studies should account for smoking exposure and other clinical confounders while measuring tissue-specific macrophage functions, rather than extrapolating periodontal marker changes directly to systemic benefit.

Human tissue observations and experimental cell and animal studies support different parts of this account: clinical associations describe disease relevance, while functional perturbations and tissue outcomes establish effects within the tested models. Taken together, macrophages contribute not only to inflammatory amplification and tissue destruction in periodontitis but also to the resolution of inflammation, bone remodeling, and tissue repair. Rather than focusing solely on the M1/M2 balance, changes in macrophage functional responses and population composition over the course of disease may help explain persistent inflammation and inadequate tissue repair. Whether these changes reflect transitions within existing macrophages or changes in recruitment, survival, and subset abundance remains incompletely resolved. Accordingly, macrophage-targeted interventions should extend beyond simply suppressing pro-inflammatory responses to restoring specific macrophage functions that promote inflammation resolution and support tissue regeneration.

## 5 Therapeutic strategies modulating macrophage responses and function

Macrophage-directed treatment should be evaluated according to both the experimental setting and the outcome measured. Human observational data, animal disease or repair models, cell-based mechanistic assays, and patient intervention studies provide different levels of evidence. Within these settings, changes in macrophage-associated markers or transcriptional profiles, directly measured cellular functions, periodontal tissue outcomes, and patient-level efficacy answer distinct questions. Mitophagy and efferocytosis assays provide functional evidence beyond marker shifts (41, 49), whereas changes in marker expression or tissue outcomes alone do not demonstrate restoration of macrophage functional plasticity or a transition between functional states.

The same distinctions guide interpretation throughout this section and in **Tables 1**–**4**. Human tissue associations establish disease relevance, cell-based perturbations test mechanisms within the specified experimental system, and animal studies assess tissue outcomes in the stated model. A therapeutic effect on one measured macrophage function can be biologically meaningful without constituting coordinated functional reprogramming. The tables therefore summarize the reported experimental setting, macrophage-related readouts, and periodontal outcomes separately, rather than treating all treatment-associated changes as evidence of functional state transitions.

**Table 1:** Representative pharmacological and bioactive molecule-based interventions targeting macrophage function in periodontitis.

Intervention	Type or source	Evidence source and experimental system	Dosing regimen	Primary mechanism of action	Reported macrophage readouts	Periodontal tissue outcomes	Ref.
Dipyridamole	Nonselective phosphodiesterase inhibitor	Experimental *in vivo* model; Cell-based studies. Rat ligature-induced periodontitis model; RAW264.7 cells stimulated with P. gingivalis LPS	Oral administration at 20 mg/kg/day for 14 days; 2 or 10 μmol/L *in vitro*	PKA and PKG signaling	Reduced pro-inflammatory macrophage-associated readouts	Reduced macrophage infiltration, osteoclast formation, and alveolar bone resorption; gingival Il1b, Il6 and Tnfa transcript reductions were not statistically significant	(71)
Rosiglitazone	Thiazolidinedione PPAR-γ agonist	Experimental *in vivo* model. Rat ligature-induced periodontitis model	Intraperitoneal administration at 5 or 10 mg/kg/day for 2 weeks	PPAR-γ-mediated immunomodulation	Reduced M1/M2-associated marker ratio, with more pronounced repair-associated features at the lower dose	Reduced inflammatory cell infiltration, osteoclast formation, and alveolar bone resorption	(76)
IL-37	Recombinant anti-inflammatory cytokine	Experimental *in vivo* model; Cell-based studies. Mouse ligature-induced periodontitis model; RAW264.7 cells stimulated with LPS or IL-4	Intrasulcular injection at 20 μg/mL (50 μL); 0.1 ng/mL *in vitro*	NLRP3 inflammasome activation and pyroptosis-related responses	Attenuated pro-inflammatory features and enhanced repair-associated features	Reduced resorption of alveolar bone, cementum, and dentin	(72)
Sema3A-Fc	Recombinant Sema3A fusion protein	Experimental *in vivo* model; Cell-based studies. Mouse model of periodontal inflammation induced by local LPS injection; RAW264.7 cells stimulated with LPS and IFN-γ	Local gingival administration at 1 mg/kg; 1 μg/mL *in vitro*	PI3K/AKT/mTOR-related signaling	Increased repair-associated macrophage features under inflammatory stimulation	Reduced inflammatory cell infiltration and improved periodontal ligament organization	(77)
DMF	Fumarate-based immunomodulatory agent	Experimental *in vivo* model; Cell-based studies. Mouse ligature-induced periodontitis model; RAW264.7 cells stimulated with P. gingivalis LPS and IFN-γ	Oral gavage at 50, 100 or 150 mg/kg/day, starting one week before ligation; 20, 40 or 60 μmol/L *in vitro*	TUFM-mediated mitophagy	Enhanced mitophagy and attenuated pro-inflammatory macrophage-associated features	Reduced inflammation, osteoclast formation, and alveolar bone resorption	(41)
Nell-1	Recombinant neural epidermal growth factor-like 1 (NELL1)	Experimental *in vivo* model; Cell-based studies. Rat ligature-induced periodontitis model; RAW264.7 cells stimulated with P. gingivalis LPS or Escherichia coli LPS	Local gingival injection of 50 μL at 2 μg/mL; 200 or 500 ng/mL *in vitro*	JNK/MAPK-related signaling	Reduced pro-inflammatory features and increased the M2/M1-associated marker ratio	Attenuated alveolar bone resorption and periodontal fiber destruction	(78)
Apabetalone	Selective BET protein inhibitor	Experimental *in vivo* model; Cell-based studies. Mouse ligature-induced periodontitis model; LPS-stimulated RAW264.7 cells and bone marrow-derived macrophages (BMDMs)	Local administration at 5 mg/kg; 2–20 μmol/L *in vitro*	BRD2 and immunometabolic reprogramming	Attenuated pro-inflammatory macrophage-associated features and restored oxidative metabolism	Reduced inflammatory cell infiltration, osteoclast formation, and alveolar bone resorption, while improving periodontal ligament architecture	(43)
Eldecalcitol	Active vitamin D analog	Experimental *in vivo* model; Cell-based studies. Diabetic rat models of periodontitis and inflammation resolution; high-glucose-stimulated RAW264.7 cells co-cultured with apoptotic neutrophils	Oral administration at 0.25 μg/kg three times per week; *in vitro* dose not reported	STIM1/Orai1-mediated store-operated calcium entry (SOCE) and efferocytosis	Restored macrophage efferocytosis under high-glucose conditions	Promoted inflammation resolution and reduced the persistence of apoptotic cells and neutrophils, osteoclast formation, and alveolar bone resorption	(68)
Butyrate	Gut microbiota-derived short-chain fatty acid (SCFA)	Experimental *in vivo* model; Cell-based studies. Diabetic mouse model of periodontitis; THP-1-derived macrophages under inflammatory stimulation	Oral administration; 200 μmol/L *in vitro* with 6 h of pretreatment	HDAC3/STAT1-related signaling and PANoptosis-like cell death	Attenuated M1-like inflammatory readouts and PANoptosis-like cell-death responses	Attenuated inflammation and alveolar bone loss while promoting osteogenic differentiation of PDLSCs	(79)
Berberine	Isoquinoline alkaloid	Experimental *in vivo* model; Cell-based studies. Ligature-induced periodontitis models in WT and db/db mice; RAW264.7 cells stimulated with P. gingivalis LPS	Oral gavage at 100 mg/kg/day; 200 μmol/L *in vitro*	NF-κB-related signaling	Attenuated pro-inflammatory features and enhanced repair-associated features	Attenuated alveolar bone resorption in both conventional and diabetes-associated periodontitis	(80)
Sulforaphene	Naturally derived isothiocyanate from radish seeds	Experimental *in vivo* model; Cell-based studies. Mouse model combining ligature placement with local bacterial stimulation; RAW264.7 cells and bone marrow-derived macrophages (BMDMs)	Intraperitoneal administration at 20 mg/kg/day; 1–10 μmol/L *in vitro*	DCIR and p38/JNK signaling	Attenuated pro-inflammatory macrophage-associated features and enhanced repair-associated features	Reduced alveolar bone resorption and improved trabecular bone parameters	(73)
Luteolin	Natural flavonoid compound	Experimental *in vivo* model; Cell-based studies. Rat ligature-induced periodontitis model; THP-1-derived macrophages stimulated with P. gingivalis LPS or TNF-α	Local gingival injection of 30 μL at 50 μmol/L; 20 μmol/L *in vitro*	Mitochondrial dynamics and JAK2/STAT3-related signaling	Improved mitochondrial dynamics and enhanced repair-associated macrophage features	Attenuated periodontal inflammation and alveolar bone resorption	(74)
Phloretin	Dietary dihydrochalcone flavonoid	Experimental *in vivo* model; Cell-based studies. Mouse ligature-induced periodontitis model; LPS-stimulated RAW264.7 cells	Oral gavage at 50 mg/kg; 30 or 100 μmol/L *in vitro*	HIF-1α-associated glycolysis and PI3K/AKT signaling	Attenuated pro-inflammatory macrophage-associated features and suppressed HIF-1α-associated glycolytic activity	Reduced periodontal inflammation, osteoclast formation, and alveolar bone resorption	(39)

Evidence scope: all interventions in this table are represented by preclinical studies. Cell-based mechanisms and animal periodontal outcomes are separate evidence levels; human dosing or periodontal efficacy is not established by these rows. The table is illustrative and does not constitute an exhaustive clinical-trial inventory. Reported experimental doses are not clinical recommendations.

Readout interpretation: Marker or transcript changes describe state-associated features, whereas cellular assays support only the specific function measured. Tissue outcomes or changes in macrophage abundance do not by themselves demonstrate within-macrophage state transitions. Functional reprogramming should therefore be inferred only when temporally linked functional and mechanistic evidence is available.

**Table 2:** Representative nucleic acid interventions and macrophage target-validation studies in periodontitis.

Target	Regulatory level	Evidence source and experimental system	Intervention	Key mechanism	Reported macrophage readouts	Periodontal outcomes	Ref.
miR-126	miRNA	Experimental *in vivo* model; Cell-based studies. Diabetes-associated periodontitis model; RAW264.7 cells	miR-126 mimic	Targets MEKK2 and suppresses MAPK and NF-κB signaling	Attenuated pro-inflammatory readouts and enhanced repair-associated features	Attenuated alveolar bone resorption	(84)
miR-217	miRNA	Human observational data; Experimental *in vivo* model; Cell-based studies. Human gingival tissues; diabetic mouse models of periodontitis and inflammation resolution; BMDMs and THP-1-derived macrophages	Local delivery of antagomiR-217	Modulates the SIRT6–miR-216/217–DEL-1/CD36 axis	Restored macrophage efferocytosis	Improved resolution after ligature removal; did not ameliorate inflammation or bone loss during continued ligation in diabetic mice	(49)
miR-128	miRNA	Human observational data; Cell-based studies. Human gingival tissues; THP-1-derived macrophages	miR-128 mimic	Inhibits p38 MAPK phosphorylation	Reduced TNF-α production and attenuated inflammatory responses	No periodontal treatment outcome is established by the cited cell-based findings	(81)
miR-223-3p	miRNA	Human observational data; Cell-based studies. Human gingival tissues and salivary EVs; THP-1-derived macrophages	miRNA mimic or inhibitor	Targets NLRP3 and modulates caspase-1–GSDMD signaling	Suppressed NLRP3-associated inflammatory responses and pyroptotic signaling	No periodontal treatment outcome is established by the cited cell-based findings	(82)
LINC01010	lncRNA	Human observational data; Cell-based studies. Human gingival tissues; human monocyte-derived macrophages	siRNA-mediated knockdown	Modulates NF-κB-associated transcription	Attenuated inflammatory responses while impairing phagocytic and antigen-processing capacities	No periodontal treatment outcome is established by the cited cell-based findings	(85)
lncR-APDC	lncRNA	Model-based evidence. Mouse ligature-induced periodontitis; single-cell sequencing of gingival tissues	Genetic knockout or local AAV9-mediated overexpression	Modulates the Tff2-associated immune network	Loss of lncR-APDC altered macrophage abundance and the distribution of transcriptionally defined states	Deficiency exacerbated inflammation and alveolar bone resorption, whereas overexpression attenuated periodontal tissue damage	(86)
METTL3	m^6^A methyltransferase	Experimental *in vivo* model; Cell-based studies. Conventional and diabetes-associated periodontitis models; RAW264.7 cells and bone marrow-derived macrophages (BMDMs)	Knockdown or local shRNA delivery	The METTL3–m^6^A–IGF2BP3 axis maintains NLRP3 mRNA stability	Suppressed NLRP3 inflammasome activation and macrophage pyroptosis	Attenuated periodontal inflammation and alveolar bone loss	(87)
Kdm6a	Histone demethylase	Experimental *in vivo* model. Circadian disruption-associated periodontitis model	Myeloid cell-specific conditional knockout	Modulates H3K27me3 and inflammatory gene transcription	Reduced pro-inflammatory gene expression and inflammatory responses	Reduced osteoclast formation and alveolar bone resorption	(88)
USP7	Deubiquitinase	Human observational data; Experimental *in vivo* model; Cell-based studies. Single-cell transcriptomic data from human gingival tissues; mouse ligature-induced periodontitis model; RAW264.7 cells	Lentiviral knockdown or pharmacological inhibition with P5091	P5091-associated changes in NF-κB p65 and STAT6 phosphorylation; inhibitor specificity remains a limitation	Knockdown reduced markers associated with both programs. P5091 reduced M1-associated markers and increased Mrc1 and Il10 transcripts and CD206 protein, but reduced Arg1 mRNA without significantly changing ARG1 protein. See Section 5.1.	No periodontal treatment outcome is established by the cited cell-based findings	(75)
UCHL1	Deubiquitinase	Experimental *in vivo* model; Cell-based studies. Mouse ligature-induced periodontitis model; RAW264.7 cells	Lentiviral knockdown, LDN-57444 treatment, or LDN@GelMA-mediated delivery	Modulates mitochondrial dysfunction and endoplasmic reticulum stress	Reduced inflammatory mediator release and improved organelle homeostasis	LDN@GelMA delivery improved inflammation and alveolar bone outcomes in mice; this was pharmacological, not nucleic acid, delivery	(42)
Act1	Signaling adaptor protein	Experimental *in vivo* model; Cell-based studies. Mouse ligature-induced periodontitis model; murine macrophages	Macrophage-associated Act1 downregulation	Enhances TNF and NF-κB signaling	Promoted inflammatory mediator expression and macrophage migration	Exacerbated periodontal inflammation and alveolar bone loss	(89)
LAPTM5	Lysosome-associated transmembrane protein	Human observational data; Cell-based studies. Human single-cell and spatial transcriptomic datasets; murine and human macrophages	siRNA-mediated knockdown	Ferroptosis-associated readouts; the detailed link to lysosomal iron handling remains unresolved	Knockdown reduced Fe²^+^ accumulation and lipid peroxidation	Improved fibroblast proliferation and migration and reduced inflammatory responses in co-culture; no *in vivo* treatment validation	(83)
BPIFA1	Host defense protein	Human observational data; Experimental *in vivo* model. Human periodontal tissues; Bpifa1-knockout mouse model of periodontitis	Systemic gene knockout	Regulates oxidative stress and NF-κB/IκB signaling	Deficiency enhanced pro-inflammatory readouts and reduced repair-associated features	Exacerbated periodontal inflammation and structural tissue damage	(90)
RGS12	Regulator of G protein signaling	Experimental *in vivo* model; Cell-based studies. Mouse ligature-induced periodontitis model; RAW264.7 cells and bone marrow-derived macrophages (BMDMs)	Conditional deletion, knockdown, or overexpression	Regulates inflammatory transcription, cell migration, and osteoclast differentiation	Deletion or knockdown reduced pro-inflammatory responses and osteoclastogenic functions	Reduced inflammatory cell infiltration, osteoclast formation, and alveolar bone destruction	(91)
A20/TNFAIP3	Negative regulator of inflammation	Experimental *in vivo* model; Cell-based studies. P. gingivalis-associated ligature-induced periodontitis model; THP-1-derived macrophages	Overexpression or knockdown	Suppresses NLRP3–caspase-1 inflammasome signaling	A20 overexpression attenuated pro-inflammatory readouts, whereas knockdown produced the opposite effects	Reduced inflammation, osteoclast formation, and alveolar bone resorption	(92)
AZGP1	Secreted glycoprotein	Experimental *in vivo* model; Cell-based studies. Conventional and diabetes-associated periodontitis models; bone marrow-derived macrophages (BMDMs) and THP-1-derived macrophages	Overexpression or genetic knockout	Activates NLRP3–caspase-1 and NF-κB/MAPK signaling	Promoted pro-inflammatory readouts and macrophage pyroptosis	Exacerbated periodontal inflammation, osteoclastic activity, and alveolar bone loss	(93)
OGT	O-GlcNAc transferase	Experimental *in vivo* model; Cell-based studies. Diabetes-associated periodontitis model; RAW264.7 cells	Knockdown or pharmacological inhibition	Promotes p38 O-GlcNAcylation and MAPK activation	Attenuated pro-inflammatory readouts and enhanced repair-associated features	Attenuated alveolar bone destruction and improved trabecular bone parameters	(94)
ENTR1/SDCCAG3	Intracellular trafficking regulator	Experimental *in vivo* model; Cell-based studies. Mouse ligature-induced periodontitis model; RAW264.7 cells	Overexpression or siRNA-mediated knockdown	Enhances AMPK phosphorylation	Attenuated pro-inflammatory readouts and enhanced repair-associated features	Attenuated periodontal inflammation and alveolar bone loss	(95)
TRPV1	Ion channel	Experimental *in vivo* model; Cell-based studies. P. gingivalis-associated ligature-induced periodontitis model; RAW264.7 cells	Pharmacological target validation with capsaicin; not nucleic acid delivery (Section 5.1)	Activates Nrf2-mediated antioxidant signaling	Reduced ROS production and inflammatory cytokine expression	Reduced inflammatory cell infiltration, osteoclast formation, and alveolar bone loss	(96)
FBLN3/EFEMP1	Extracellular matrix protein	Human observational data; Experimental *in vivo* model; Cell-based studies. Human gingival tissues; mouse ligature-induced periodontitis model; bone marrow-derived macrophages (BMMs) and RAW264.7 cells	Knockdown, overexpression, or local RNA interference	Activates EGFR–PI3K–AKT signaling	FBLN3 inhibition reduced pro-inflammatory readouts and enhanced repair-associated features	Reduced periodontal inflammation and alveolar bone resorption	(97)

Evidence scope: this table includes nucleic acid interventions and molecular-target validation. Lentiviral knockdown, knockout, and overexpression are distinguished from pharmacological probes; P5091, UCHL1 inhibitor delivery, and TRPV1 activation with capsaicin are discussed as drug interventions in Section 5.1. Human tissue expression data are observational, not therapeutic clinical evidence.

Readout interpretation: Marker or transcript changes describe state-associated features, whereas cellular assays support only the specific function measured. Tissue outcomes or changes in macrophage abundance do not by themselves demonstrate within-macrophage state transitions. Functional reprogramming should therefore be inferred only when temporally linked functional and mechanistic evidence is available.

**Table 3:** Representative studies of cell- and extracellular vesicle-based interventions in periodontitis.

Intervention strategy	Cell or vesicle source	Evidence source and experimental system	Key mechanism	Reported macrophage readouts	Periodontal outcomes	Ref.
PDLSC transplantation	Periodontal ligament stem cells (PDLSCs)	Experimental *in vivo* model; Cell-based studies. Rat periodontal defect model; bone marrow-derived macrophages (BMDMs)	Modulates the local immune microenvironment through paracrine signaling	Attenuated pro-inflammatory readouts and enhanced repair-associated features	Enhanced new bone formation and promoted cementum-like tissue formation and collagen fiber insertion	(65)
Local periodontal injection of iMSCs	Induced pluripotent stem cell-derived mesenchymal stem cells (iMSCs)	Experimental *in vivo* model; Cell-based studies. Mouse ligature-induced periodontitis model; THP-1-derived macrophages	Suppresses NF-κB activation and reduces oxidative stress	Attenuated pro-inflammatory readouts and enhanced repair-associated features	Preserved the osteogenic capacity of PDLSCs under inflammatory conditions and reduced alveolar bone loss	(98)
BMSC spheroid implantation	Three-dimensional spheroids formed by bone marrow mesenchymal stem cells (BMSCs)	Experimental *in vivo* model; Cell-based studies. Rat periodontal fenestration defect model; RAW264.7 cells	Modulates macrophage function through paracrine TGF-β1 signaling	Attenuated pro-inflammatory readouts and enhanced repair-associated features	Reduced local inflammation and promoted osteogenic differentiation and bone defect repair	(108)
Adoptive transfer of B10 cells	IL-10-producing regulatory B cells	Experimental *in vivo* model; Cell-based studies. Mouse model of experimental periodontitis; macrophage co-culture system	Modulates macrophage function and promotes resolvin D2 (RvD2) production, efferocytosis, and autophagy	Increased RvD2 production, efferocytosis, and autophagy, with reduced pro-inflammatory readouts	Reduced inflammation, osteoclast activation, and alveolar bone resorption, with these effects being partially macrophage dependent	(99, 100)
Local injection of IL-4 backpack-loaded macrophages	Bone marrow-derived macrophages (BMDMs) carrying surface-bound sustained-release IL-4 backpacks	Experimental *in vivo* model; Cell-based studies. Mouse model of experimental periodontitis; *in vitro* inflammatory model	Provides sustained IL-4 release through cell-surface backpacks	Maintained repair-associated macrophage features during inflammatory exposure	Suppressed the progression of experimental periodontitis	(101)
PZ-GMs hydrogel-mediated delivery	Intracellularly gelated macrophages loaded with phytic acid and Zn²^+^	Experimental *in vivo* model; Cell-based studies. Mouse model of periodontitis; *in vitro* pathogen and toxin assays	Uses macrophage membranes to recognize and neutralize pathogenic stimuli, combined with the antibacterial activity of Zn²^+^ and the mineralization-promoting effects of phytic acid	Preserved pathogen-recognition and inflammatory mediator-neutralizing functions	Eliminated pathogens, toxins, and inflammatory mediators, improved the oral microbial environment, and promoted alveolar bone regeneration	(109)
BMSC-sEV hydrogel-mediated delivery	Small extracellular vesicles derived from bone marrow mesenchymal stem cells (BMSC-sEVs)	Experimental *in vivo* model; Cell-based studies. Human periodontal ligament cells (hPDLCs); rat model of experimental periodontitis	Modulates the OPG–RANKL–RANK axis and TGF-β1 expression while improving the local immune microenvironment	Increased repair-associated macrophage features and the M2/M1-associated marker ratio in treated periodontal tissues	Promoted hPDLC migration, proliferation, and osteogenic differentiation while reducing inflammatory cell infiltration, collagen degradation, and alveolar bone resorption	(110)
L-D-sEV hydrogel-mediated delivery	Small extracellular vesicles derived from LPS-preconditioned dental follicle cells (L-D-sEVs)	Experimental *in vivo* model. hPDLCs under inflammatory conditions; rat and mouse models of experimental periodontitis	Reduces JNK and p38 phosphorylation and the RANKL/OPG ratio, thereby modulating macrophage and osteoclast responses	Attenuated pro-inflammatory readouts and enhanced repair-associated features	Reduced PDLC apoptosis and osteoclast activity, attenuated alveolar bone loss, and promoted periodontal tissue regeneration	(111, 112)
GMSC-EV treatment	EVs derived from human gingival mesenchymal stem cells (GMSC-EVs)	Experimental *in vivo* model; Cell-based studies. Rat model of periodontitis; THP-1-derived macrophages stimulated with P. gingivalis LPS	Suppresses HIF-1α signaling and shifts macrophage metabolism from glycolysis toward oxidative phosphorylation	Attenuated pro-inflammatory readouts and enhanced repair-associated metabolic features	Improved the periodontal inflammatory microenvironment and promoted tissue repair	(103)
Local gingival injection of EVs-ASP	Aspirin-loaded extracellular vesicles derived from periodontal ligament stem cells (PDLSCs)	Experimental *in vivo* model; Cell-based studies. Mouse model of LPS-induced periodontitis; LPS-stimulated bone marrow-derived macrophages (BMDMs)	Enhances oxidative phosphorylation while suppressing glycolysis	Attenuated pro-inflammatory readouts and enhanced repair-associated metabolic features	Reduced alveolar bone loss and partially restored alveolar bone height	(113)
Local injection of hPDLSC-ApoEVs	Apoptotic extracellular vesicles derived from human periodontal ligament stem cells (hPDLSC-ApoEVs)	Experimental *in vivo* model; Cell-based studies. THP-1-derived macrophages; hPDLSC co-culture system; mouse ligature-induced periodontitis model	Modulates the NF-κB/S100A9 signaling axis	Enhanced CD163-, IL-10-, and Arg-1-associated reparative features	Improved the inflammatory microenvironment, promoted osteogenic differentiation of hPDLSCs, and enhanced alveolar bone regeneration	(104)
Hyaluronic acid-assisted delivery of PDLSC-ApoEVs	Apoptotic extracellular vesicles derived from periodontal ligament stem cells (PDLSC-ApoEVs)	Experimental *in vivo* model; Cell-based studies. LPS-stimulated RAW264.7 cells; rat model of experimental periodontitis	Transfers mitochondrial components, including mtDNA, to macrophages, thereby restoring mitochondrial membrane potential and ATP production	Reduced pro-inflammatory mediator expression, improved mitochondrial homeostasis, and enhanced repair-associated features	Improved the periodontal immune microenvironment, reduced tissue destruction, and promoted periodontal tissue regeneration	(105)
HBCH/P-sEVs composite repair system	Small extracellular vesicles derived from human periodontal ligament stem cells (hPDLSC-sEVs)	Human observational data; Experimental *in vivo* model; Cell-based studies. Human gingival tissues; macrophages stimulated with P. gingivalis LPS and ATP; rat model of periodontitis-associated bone defects	Suppresses NF-κB–NLRP3–caspase-1–GSDMD signaling and enables local delivery through a thermosensitive hydrogel	Suppressed macrophage pyroptosis and inflammatory mediator release	Promoted hPDLSC proliferation and osteogenic differentiation, attenuated local inflammation, and enhanced alveolar bone regeneration	(114)
Local delivery of GMSC-sEV composite microparticles	Minocycline-loaded PLGA microparticles functionalized with GMSC-derived small extracellular vesicles (sEVs)	Experimental *in vivo* model; Cell-based studies. Rat model of periodontal disease; monocytes, macrophages, and T cells	Enables MMP-responsive release of sEVs and sustained release of minocycline to suppress bacterial biofilms	Reduced pro-inflammatory cytokine secretion by monocytes and macrophages	Reduced bacterial burden and local inflammation while promoting periodontal tissue regeneration	(115)
Local delivery of M2-EVs	Extracellular vesicles derived from repair-associated M2-like macrophages	Human observational data; Experimental *in vivo* model. Human periodontal tissues; mouse model of inflammation resolution following ligature removal; neutrophil analyses and single-cell sequencing	Associated with an Anxa1^hi^ immature neutrophil state; pseudotime analysis implicates Acvrl1/Fpr2, while CellChat and tissue staining suggest OSM-linked stromal communication	Recipient macrophage function was not directly assessed. The intervention primarily affected macrophage–neutrophil communication.	Reduced alveolar bone loss and osteoclastic activity while enhancing osteogenic and angiogenic repair	(116)
Treatment with M2-like macrophage-derived EVs	EVs derived from IL-4- and IL-13-induced M2-like macrophages	Cell-based studies. *In vitro* osteoclast differentiation model induced by RANKL and M-CSF	Delivers miR-1227-5p to target OSCAR	Recipient macrophage function was not directly evaluated	Inhibited osteoclast differentiation; No periodontal treatment outcome is established by the cited cell-based findings	(106)
sh-Ckip-1-EV treatment and ectopic implantation	Engineered EVs derived from Ckip-1-silenced macrophages	Experimental *in vivo* model; Cell-based studies. P. gingivalis-stimulated macrophages; OCCM-30 cells; mouse subcutaneous ectopic implantation model	Delivers let-7f-5p to target Ckip-1 and activate PGC-1α-dependent mitochondrial biogenesis	Attenuated pro-inflammatory readouts and enhanced repair-associated features in donor macrophages	Restored P. gingivalis-impaired cementoblast mineralization and promoted ectopic formation of cementum-like tissue	(117)
ICG@M2-EVs combined with near-infrared irradiation	ICG-loaded EVs derived from hypoxia-preconditioned M2-like macrophages	Experimental *in vivo* model; Cell-based studies. Rat model of P. gingivalis-induced periodontitis; THP-1-derived macrophages; P. gingivalis and biofilm assays	ICG mediates photodynamic antibacterial activity, while the EVs alleviate oxidative stress and modulate macrophage function	Attenuated pro-inflammatory readouts and enhanced repair-associated features	Reduced infection, inflammation, and oxidative stress while promoting alveolar bone regeneration	(118)
Local treatment with M2-EVs	EVs derived from repair-associated M2-like macrophages	Experimental *in vivo* model; Cell-based studies. Bone marrow mesenchymal stem cells (BMSCs); BMDM-derived osteoclasts; mouse model of periodontitis	Delivers IL-10 mRNA and activates IL-10/IL-10R signaling in BMSCs and BMDMs	Inhibited the differentiation of BMDMs into osteoclasts	Promoted osteogenic differentiation of BMSCs while reducing osteoclast formation and alveolar bone resorption	(61)
Local injection of miR-23b-EV	Engineered EVs derived from miR-23b-overexpressing HEK293T cells	Experimental *in vivo* model; Cell-based studies. LPS-stimulated RAW264.7 cells; rat ligature-induced periodontitis model	Delivers miR-23b and suppresses TAB2-, TAB3-, and IKKα-mediated NF-κB signaling	Reduced inflammatory cytokine release and other pro-inflammatory readouts while enhancing repair-associated features	Reduced inflammatory cell infiltration and osteoclast formation, thereby attenuating alveolar bone resorption and periodontal tissue damage	(119)
Local injection of LEVs^Tet-PKM2^@TA	Large extracellular vesicles derived from HEK293T cells, enriched with tetrameric PKM2 and modified with tannic acid	Human observational data; Experimental *in vivo* model; Cell-based studies. Human gingival tissues; LPS-stimulated RAW264.7 cells; mouse ligature-induced periodontitis model	Tannic acid facilitates lysosomal escape and delivery of tetrameric PKM2, restoring pyruvate flux into the TCA cycle and oxidative phosphorylation	Restored oxidative metabolism, attenuated pro-inflammatory readouts, and enhanced repair-associated features	Reduced inflammatory cell infiltration and alveolar bone loss, improved trabecular bone architecture, and promoted periodontal tissue repair	(107)

EV nomenclature: EV is used as the general term in this table. Small and large EVs denote operational size categories, whereas apoptotic EVs denote an apoptotic donor-cell origin. These names do not by themselves establish a biogenesis pathway.

Evidence scope: Human tissue observations, cell-based studies, animal periodontitis models, and periodontal defect repair models represent different evidence sources and do not constitute patient intervention evidence.

Readout interpretation: Marker or transcript changes describe state-associated features, whereas cellular assays support only the specific function measured. Tissue outcomes or changes in macrophage abundance do not by themselves demonstrate within-macrophage state transitions. Functional reprogramming should therefore be inferred only when temporally linked functional and mechanistic evidence is available.

**Table 4:** Representative biomaterials and local delivery systems targeting macrophages in periodontitis.

Material system	Major components	Evidence source and experimental system	Material or delivery features	Mechanisms of macrophage modulation	Major periodontal outcomes	Ref.
Q@MPDA nanoparticles	Quercetin-loaded mesoporous polydopamine	Experimental *in vivo* model; Cell-based studies. Inflammatory macrophages; mouse model of periodontitis	Enhances gingival adhesion and local retention, enabling sustained delivery of quercetin	Suppresses glycolysis, enhances oxidative phosphorylation, and reduces mitochondrial ROS, accompanied by reduced pro-inflammatory readouts and enhanced repair-associated features	Attenuated periodontal inflammation, osteoclast formation, and alveolar bone resorption	(120)
GOE1 hydrogel	Self-assembled nanoparticles composed of chlorhexidine and epigallocatechin gallate (EGCG), GelMA, and oxidized hyaluronic acid	Experimental *in vivo* model; Cell-based studies. RAW264.7 cells and gingival fibroblasts; diabetic rat model of periodontitis	Injectable and tissue-adhesive, with sustained release of antibacterial and antioxidant components	Scavenges ROS and modulates IL-17-associated inflammatory signaling, accompanied by reduced pro-inflammatory readouts and enhanced repair-associated features	Inhibited bacterial growth and reduced inflammatory cell infiltration, collagen degradation, and alveolar bone loss	(121)
8AGPB supramolecular hydrogel	8-Aminoguanosine (8AG) and 1,4-benzenediboronic acid	Experimental *in vivo* model; Cell-based studies. LPS-stimulated macrophages and gingival fibroblasts; mouse model of periodontitis	Exhibits shear-thinning, self-healing, and injectable properties and releases 8AG *in situ* upon degradation	Modulates second messengers, including Ca²^+^ and cAMP, as well as MAPK and NF-κB signaling, thereby reducing ROS accumulation and pro-inflammatory mediator release	Reduced inflammatory cell infiltration, osteoclastic activity, and periodontal tissue destruction	(122)
PFD/CBD octopus-inspired adhesive nanomicelles	PBA-Fu-DOCA nanomicelles loaded with cannabidiol (CBD)	Experimental *in vivo* model; Cell-based studies. Macrophages, neutrophils, and bone marrow mesenchymal stem cells (BMSCs); mouse model of periodontitis	Enhances adhesion to the periodontal mucosa and promotes uptake by inflammatory cells through scavenger receptor A (SR-A), while enabling sustained drug release	Reduced ROS levels in macrophages and neutrophils, suppressed NET formation, and enhanced repair-associated macrophage features	Reduced inflammation, NET accumulation, and osteoclastic activity while promoting alveolar bone regeneration	(123)
TPP-SAT natural enzyme cascade nanogel	Triphenylphosphonium (TPP), superoxide dismutase (SOD), and catalase (CAT)	Experimental *in vivo* model; Cell-based studies. Macrophages, vascular endothelial cells, and fibroblasts; rat model of periodontitis	TPP mediates mitochondrial targeting, while the SOD/CAT enzyme cascade scavenges ROS and generates oxygen	Scavenged mitochondrial ROS, alleviated tissue hypoxia, and activated Nrf2 signaling, accompanied by changes in pro-inflammatory and repair-associated macrophage readouts	Attenuated inflammation and alveolar bone resorption while promoting angiogenesis and periodontal tissue repair	(124)
SF-HA@TA-Mn nanomodulator	Silk fibroin nanoparticles, a hydroxyapatite layer, and TA-Mn coordination units	Experimental *in vivo* model; Cell-based studies. Macrophages and human periodontal ligament stem cells (hPDLSCs); rat model of periodontitis	Tannic acid (TA) facilitates mitochondrial targeting, Mn enhances antioxidant activity, and SF-HA provides osteoconductive support	Scavenges mitochondrial ROS, preserves mitochondrial homeostasis, downregulates the JAK2–STAT1–ASS1 axis, and modulates arginine metabolism together with TCA cycle and purine metabolic pathways	Attenuated inflammation and oxidative damage, restored osteogenic differentiation of hPDLSCs, and promoted alveolar bone regeneration	(125)
MBG@L-Arg	L-arginine-functionalized mesoporous bioactive glass	Experimental *in vivo* model; Cell-based studies. Macrophages, MC3T3-E1 cells, and human periodontal ligament stem cells (hPDLSCs); rat model of periodontitis	Scavenges ROS and provides sustained release of nitric oxide (NO) and bioactive ions	Suppresses NF-κB signaling and activates Nrf2, accompanied by reduced pro-inflammatory readouts and enhanced repair-associated features	Reduced periodontal tissue destruction and promoted osteogenesis and alveolar bone repair through NO-related signaling	(126)
MPB-BA	Baicalein-loaded mesoporous Prussian blue	Experimental *in vivo* model; Cell-based studies. Periodontal pathogens and macrophages; experimental periodontitis model	Combines baicalein delivery, ROS scavenging, and near-infrared photothermal antibacterial activity	Activates Nrf2 and suppresses NF-κB signaling, thereby reducing oxidative stress and pro-inflammatory macrophage responses	Reduced bacterial burden, inflammation, and alveolar bone destruction	(127)
LMCNDs engineered nanodecoys	LL-37-functionalized macrophage membrane-coated cascade system containing L-amino acid oxidase (LAAO) and hollow MnO_2_	Experimental *in vivo* model; Cell-based studies. Periodontal pathogens, macrophages, and osteoblasts; mouse model of periodontitis	The macrophage membrane enhances pathogen recognition and tissue retention, while the enzymatic cascade alleviates hypoxia and oxidative stress	Promotes Nrf2 nuclear translocation, reduces oxidative stress, and alters macrophage inflammatory and repair-associated readouts	Enhanced clearance of periodontal pathogens, attenuated inflammation, and promoted alveolar bone regeneration	(128)
MM@PPT composite microspheres	Activated macrophage membrane-coated, PLL-modified PLGA/β-TCP microspheres	Experimental *in vivo* model; Cell-based studies. Macrophages, osteoclasts, and bone marrow mesenchymal stem cells (BMSCs); rat model of periodontitis	Membrane-associated receptors sequester inflammatory cytokines, while PLGA/β-TCP provides local structural and osteoconductive support	Neutralizes TNF-α and IL-6, reduces pro-inflammatory macrophage responses and osteoclast differentiation, and improves immune–osteogenic coupling	Reduced alveolar bone resorption and promoted alveolar bone regeneration	(129)
CPS@BA charge-adaptive nanoparticles	BAPTA-AM-loaded sialic acid-modified CMCS–PEI copolymer	Experimental *in vivo* model; Cell-based studies. Neutrophils, neutrophil extracellular traps (NETs), and macrophages; periodontitis models in wild-type and Pad4-deficient mice	Undergoes charge switching in the acidic inflammatory microenvironment to capture NETs and enable responsive release of a Ca²^+^ chelator	Suppresses PAD4-dependent NET formation, disrupts CXCL3–CXCR2-mediated NET–macrophage crosstalk, and improves macrophage mitochondrial function	Reduced inflammatory cell infiltration, osteoclast formation, and alveolar bone resorption	(130)
Ac2-26/OGP smart hydrogel	ROS- and pH-responsive GPPG hydrogel loaded with Ac2–26 and osteogenic growth peptide (OGP)	Experimental *in vivo* model; Cell-based studies. Inflammatory macrophages and osteoblasts; rat extraction socket model of diabetes-associated periodontitis	Enables stimuli-responsive, sequential release of the anti-inflammatory peptide Ac2–26 and osteogenic growth peptide OGP	Suppresses macrophage pyroptosis and inflammatory mediator release through the ANXA1–NLRP3–caspase-1–GSDMD axis	Attenuated diabetes-associated inflammation and promoted new bone formation within the extraction socket	(131)
CCL2 sustained-release microparticles	CCL2-loaded PLGA microparticles	Experimental *in vivo* model; Cell-based studies. RAW264.7 cells and bone marrow-derived macrophages; mouse models of periodontitis induced by P. gingivalis infection and ligature placement	Provides sustained CCL2 release and locally recruits endogenous monocytes and macrophages	Enhances macrophage chemotaxis, increases the proportion of CD206-associated repair-related cells, and modulates IL-1RA and RANKL expression	Reduced osteoclast numbers and alveolar bone loss	(59)
TPG/PLGA/PCL nanofibrous membrane	Tea polyphenol-functionalized graphene oxide, PLGA, and PCL	Experimental *in vivo* model; Cell-based studies. RAW264.7 cells and human periodontal ligament stem cells (hPDLSCs); rat model of periodontal bone defects	Serves as a guided tissue regeneration (GTR) barrier membrane to maintain regenerative space while improving material hydrophilicity and cell adhesion	Suppresses PI3K/AKT and NF-κB signaling, thereby attenuating pro-inflammatory features and enhancing repair-associated features	Promoted regeneration of alveolar bone, periodontal ligament, and directionally organized collagen fibers	(132)
PLN periodic lamellar nanofibers	PCL nanofibers with a periodic lamellar topography	Experimental *in vivo* model; Cell-based studies. LPS-stimulated macrophages and bone marrow mesenchymal stem cells (BMSCs); mouse model of periodontitis	Provides drug-free physical cues through surface nanotopography	Activates integrin–RhoA–YAP signaling, with YAP interacting with IKK-α to suppress NF-κB signaling and enhance repair-associated macrophage features	Promoted BMSC migration and osteogenesis while reducing inflammation-associated bone loss	(133)
GelMA/t-BTO piezoelectric hydrogel	Tilapia-derived GelMA hydrogel loaded with tetragonal BaTiO_3_ (t-BTO) nanoparticles	Experimental *in vivo* model; Cell-based studies. PDLSCs and macrophages under inflammatory conditions; rat model of periodontitis-associated bone defects	Mechanically responsive piezoelectric system; ultrasound was used in the cell experiments	*In vitro* piezoelectric stimulation increased Arg-1 without an obvious iNOS change. Treated rat defects showed lower iNOS and higher CD206. Bioenergetic restoration was examined primarily in PDLSCs	Attenuated local inflammation and promoted regeneration of periodontal bone defects	(134)
P@PCH bilayer nanofibrous membrane	PCL barrier layer and an HMB-Ca-loaded PVA/COS functional layer	Experimental *in vivo* model; Cell-based studies. Macrophages and rat bone marrow mesenchymal stem cells (rBMSCs); rat model of periodontal defects	The PCL layer prevents epithelial ingrowth, COS modulates the local immune response, and HMB-Ca releases Ca²^+^ to promote stem cell recruitment	Reduced TNF-α and iNOS expression while enhancing CD206-, IL-10-, TGF-β-, VEGF-, and OSM-associated reparative signals	Reduced inflammatory cell infiltration, promoted endogenous stem cell recruitment, and enhanced repair of periodontal bone defects	(135)
HIPPE-QU injectable emulsion gel	Gelatin nanoparticles, copper-doped hydroxyapatite, and quercetin	Experimental *in vivo* model; Cell-based studies. Macrophages, rat bone marrow mesenchymal stem cells (rBMSCs), and gingival fibroblasts; rat model of periodontitis	Exhibits injectability, shear-thinning behavior, and acid-responsive sustained release	Suppresses TLR-, AMPK-, and HIF-1-associated glycolytic signaling, restores mitochondrial membrane potential and ATP production, and enhances repair-associated macrophage features	Reduced inflammation and osteoclastic activity while improving alveolar bone volume and periodontal tissue architecture	(136)
A/CQ-ML@Gel smart hydrogel	CS–P407–PNIPAM hydrogel loaded with AgNPs, a quercetin microemulsion, and a CAPE lipid complex	Experimental *in vivo* model; Cell-based studies. P. gingivalis, inflammatory macrophages, and periodontal ligament stem cells (PDLSCs); rat model of periodontitis	Exhibits temperature- and pH-responsive properties, enabling programmed release of quercetin and CAPE	Activates PINK1/Parkin-dependent mitophagy, promotes clearance of damaged mitochondria, and reduces ROS accumulation	Exerted antibacterial and anti-inflammatory effects while promoting alveolar bone regeneration	(137)
CFMD thermosensitive nanocomposite hydrogel	Chitosan–PF127 hydrogel loaded with folic acid-modified mesoporous bioactive glass and doxycycline	Experimental *in vivo* model; Cell-based studies. Macrophages and human periodontal ligament stem cells (hPDLSCs); rat model of periodontitis	Undergoes *in situ* gelation at body temperature, with folic acid-mediated targeting of activated macrophages and sustained release of therapeutic agents and osteogenic ions	Reduced iNOS and inflammatory mediators, increased CD206, and supported mitochondrial function; oxidative-phosphorylation enrichment was detected in periodontal tissue transcriptomes, not as macrophage-specific metabolic flux	Suppressed periodontal pathogens and inflammation, improved trabecular bone architecture, and promoted alveolar bone regeneration	(138)
AA@GO composite hydrogel	Asiatic acid, graphene oxide, sodium alginate, and carboxymethyl chitosan	Experimental *in vivo* model; Cell-based studies. Macrophages under high-glucose conditions; mouse model of diabetes-associated periodontitis	Enhances local encapsulation and sustained release of asiatic acid	Enhances mitophagy, reduces ROS accumulation and damaged mitochondria, restores mitochondrial function, and modulates pro-inflammatory and repair-associated macrophage responses	Attenuated diabetes-associated periodontal inflammation and alveolar bone damage	(139)

Evidence scope: the listed platforms are experimental delivery or tissue-repair systems. Cell assays, animal disease models, and bone-defect models answer different questions. Reduced inflammatory markers or improved bone measurements do not alone establish macrophage specificity, complete periodontal regeneration, or clinical efficacy.

Readout interpretation: Marker or transcript changes describe state-associated features, whereas cellular assays support only the specific function measured. Tissue outcomes or changes in macrophage abundance do not by themselves demonstrate within-macrophage state transitions. Functional reprogramming should therefore be inferred only when temporally linked functional and mechanistic evidence is available.

### 5.1 Pharmacological and bioactive molecule-based interventions

The pharmacological studies report tissue protection alongside variable changes in inflammatory markers. Dipyridamole reduced bone loss and osteoclastogenesis in ligature-induced rat periodontitis, while RAW264.7 assays implicated PKA/PKG signaling (71). However, gingival Il1b, Il6, and Tnfa transcript reductions were not statistically significant (71), indicating that bone protection need not coincide with uniform suppression of inflammatory readouts. Similarly, reduced inflammasome-associated signals accompanied periodontal protection in the IL-37 study using macrophage cultures and a mouse ligature model (72). Together, these findings support preclinical activity, while leaving the relative contribution of individual macrophage pathways unresolved.

Functional rescue provides a complementary basis for evaluating these agents. In a regimen initiated one week before ligature placement, dimethyl fumarate improved macrophage mitochondrial quality control and reduced periodontal destruction in mice; attenuation of its effects after TUFM knockdown *in vitro* supports a role for this pathway in the tested cells (41). Eldecalcitol linked restoration of store-operated calcium entry to improved efferocytosis in high-glucose macrophage assays, alongside reduced bone loss in diabetic rats (68). These experiments provide evidence of recovery in specific measured macrophage functions rather than evidence of a complete transition between macrophage states. These studies connect a defined cellular defect with tissue benefit, but do not establish that macrophages account for all *in vivo* effects or that the same mechanism will predict patient benefit. We consider treatment of established disease a more relevant next test than further confirmation of a preventive effect. In particular, the DMF pretreatment design (41) leaves its efficacy after disease establishment unresolved; a delayed-start arm would test that question more directly than further increases in polarization-marker measurements.

The natural compounds reviewed here act through different pathways. Sulforaphene studies used DCIR knockdown and overexpression to support receptor involvement (73); luteolin studies linked changes in mitochondrial dynamics and JAK2/STAT3 signaling to responses in THP-1-derived macrophages and rat periodontitis (74); phloretin combined macrophage glycolytic assays with a mouse periodontal model (39). These are distinct mechanistic examples, not independent replications of one intervention. Differences in exposure, experimental systems, and endpoints leave unresolved which pathways are necessary for tissue protection, whether effects persist after treatment, and how the agents compare under matched conditions.

Small-molecule inhibition must also be distinguished from nucleic acid delivery. P5091 reduced inflammatory markers and NF-κB p65 phosphorylation, and increased STAT6 phosphorylation, in stimulated RAW264.7 macrophages. Its effects on IL-4-associated markers were not uniform: Mrc1 and Il10 transcripts and CD206 protein increased, whereas Arg1 mRNA decreased and ARG1 protein did not change significantly. The authors also noted reported inhibition of USP47 by P5091, which limits attribution of its effects solely to USP7 (75). The same study reported that lentiviral USP7 knockdown reduced markers associated with both conventionally defined M1- and M2-like responses, illustrating that genetic depletion and pharmacological inhibition are not interchangeable. These cellular effects do not, by themselves, establish therapeutic periodontal efficacy *in vivo*. UCHL1 inhibitor delivery in a hydrogel, by contrast, was evaluated after ligature removal in experimental periodontitis and accompanied improvement in mitochondrial and endoplasmic-reticulum homeostasis in macrophage assays (42). Capsaicin-mediated TRPV1 activation also belongs to pharmacological target validation: the cited experimental study reported Nrf2-associated antioxidant responses and reduced periodontal destruction (96).

The principal translational gap is therefore the link between experimental target engagement and durable periodontal benefit. The selected studies do not establish a human periodontal dose, sustained local exposure, added benefit over conventional care, or long-term preservation of antimicrobial defense. Comparative studies with functional and tissue endpoints are needed before promising marker changes can guide agent selection. Representative agents and their experimental systems are summarized in **Table 1**.

### 5.2 Nucleic acid-based interventions and genetic target validation

miRNA-based interventions can attenuate inflammation by supplementing protective miRNAs or antagonizing aberrantly upregulated miRNAs, with some studies demonstrating recovery of specific macrophage functions. In diabetic mice, local antagomiR-217 delivery supported efferocytosis and inflammation resolution through the SIRT6–miR-216/217–DEL-1/CD36 axis after ligature removal. However, it did not ameliorate inflammation or bone loss under continued ligation, making the disease phase an important boundary of the reported benefit (49). Similarly, miR-128 mimics can suppress p38 MAPK phosphorylation and TNF-α production, suggesting a potential role for miR-128 in limiting excessive inflammatory responses (81). However, current evidence is derived primarily from human gingival tissues and THP-1-derived macrophages, and *in vivo* validation of periodontal outcomes remains lacking. In addition, miR-223-3p can directly target NLRP3, thereby modulating caspase-1–GSDMD-associated inflammasome activation and macrophage pyroptosis (82). These findings indicate that nucleic acid-based interventions can alter macrophage-associated inflammatory pathways and, in selected studies, directly affect measured cellular processes including efferocytosis, inflammasome activation, and pyroptotic cell death.

Genetic perturbation is valuable for identifying disease-relevant targets, but should be separated from a therapeutically deliverable nucleic acid intervention. USP7 knockdown reduced markers associated with both conventionally defined response programs, whereas P5091 produced a different pattern of responses (75). The comparison cautions against assuming that a small-molecule phenotype predicts the effect of gene silencing. Similarly, UCHL1 studies combined genetic manipulation with inhibitor experiments; efficacy of the inhibitor-loaded hydrogel is pharmacological delivery evidence, not proof of a nucleic acid therapy (42). LAPTM5 siRNA experiments used RAW264.7–L929 and human monocyte-derived macrophage–gingival fibroblast co-cultures. Knockdown reduced macrophage Fe²^+^ accumulation and lipid peroxidation, improved fibroblast proliferation and migration, and attenuated inflammatory responses. These cell-based findings complement human tissue and transcriptomic observations, but do not establish *in vivo* periodontal treatment efficacy or the precise molecular mediators of macrophage–fibroblast communication (83).

**Table 2** therefore distinguishes nucleic acid delivery and genetic target-validation experiments from pharmacological probes used in the same research programs. Knockout or overexpression findings identify biological dependencies; they do not by themselves demonstrate feasible therapeutic delivery, macrophage selectivity, or a safe treatment window. Particularly informative future studies would test whether a defined nucleic acid formulation preserves antimicrobial activity while improving directly measured efferocytosis or tissue-supportive function.

### 5.3 Cell- and extracellular vesicle-based therapies

Beyond nucleic acid- and molecular target-based interventions, cell-based therapies can simultaneously modulate periodontal inflammation and tissue repair through sustained paracrine signaling and intercellular interactions. Periodontal ligament stem cell (PDLSC) transplantation increases the abundance of CD163^+^ macrophages during the early phase of repair, accompanied by the formation of new bone, cementum-like structures, and collagen fibers inserting into the newly formed cementum (65). In models of active periodontitis, iMSCs suppress NF-κB activation and oxidative stress in macrophages, thereby preserving the osteogenic capacity of PDLSCs under inflammatory conditions and reducing alveolar bone loss (98). Beyond stem cell-mediated indirect immunomodulation, adoptive transfer of B10 cells can enhance the pro-resolving functions of macrophages by promoting resolvin D2 (RvD2) production, clearance of apoptotic neutrophils, and autophagy, thereby reducing inflammation and alveolar bone resorption (99, 100). When macrophages themselves are used as therapeutic carriers, maintenance of their intended response profile within the inflammatory environment becomes an important consideration. Macrophages equipped with sustained-release IL-4 “backpacks” maintained repair-associated features during inflammatory exposure and suppressed the progression of experimental periodontitis (101). Thus, the therapeutic value of cell-based approaches extends beyond simply providing regenerative cells. These strategies may also modulate local immunoregulatory and tissue-supportive responses. Because periodontal defect models and active periodontitis models represent distinct pathological processes, therapeutic outcomes should be interpreted in the context of the specific experimental model used.

Extracellular vesicle (EV) terminology is used consistently throughout the narrative and tables. Small EVs and large EVs are operational size categories, whereas exosome refers to an endosomal origin; apoptotic EVs are described by the state of the donor cell rather than size alone (102). We therefore use EV as the general term, specify small, large, or apoptotic EVs when relevant, and avoid inferring biogenesis from a preparation name.

Distinct EV preparations also target different functions. Gingival mesenchymal stromal-cell EVs altered HIF-1α-associated macrophage metabolism *in vitro* and were evaluated in rat periodontitis (103). PDLSC-derived apoptotic EVs were investigated for regulation of NF-κB/S100A9 signaling and for transfer of mitochondrial components to recipient macrophages (104, 105). Macrophage-derived EVs carrying IL-10 mRNA affected osteogenic differentiation and osteoclast formation in cell systems and reduced bone loss in mice (61). In contrast, the reported miR-1227-5p experiments assessed osteoclast differentiation *in vitro*; these cellular findings alone do not establish periodontal treatment efficacy *in vivo* (106).

Engineered large EVs enriched in tetrameric PKM2 and modified with tannic acid were designed to improve intracellular delivery and restore oxidative metabolism in inflammatory macrophages (107). Their size class and engineering strategy distinguish them from the small-EV preparations listed in **Table 3**. Across these studies, donor cells, separation methods, dose metrics, cargo manipulation, and recipient assays differ substantially. Outcomes cannot be attributed to an EV class solely from its name, nor can efficacy be compared without accounting for these differences. Product identity, purity, potency, local retention, and repeated-dose safety remain requirements for clinical development.

### 5.4 Biomaterial-mediated local delivery and immunomodulation

In the experimental delivery systems summarized in **Table 4**, fluid flow, tissue movement, and inflammatory exudation within periodontal pockets can limit the local retention and bioavailability of free drugs. Biomaterial-based delivery systems are therefore designed to prolong drug retention, enable controlled release, and enhance lesion-specific delivery. Q@MPDA exploits the adhesive properties of mesoporous polydopamine to reduce salivary clearance of quercetin and provides sustained local release, thereby increasing its effective exposure within gingival tissues (120). Similarly, to overcome rapid local drug clearance, GOE1 employs an injectable, tissue-adhesive hydrogel to form an *in situ* delivery system within the periodontal pocket, enabling sustained release of chlorhexidine and epigallocatechin gallate and thereby prolonging their antibacterial, antioxidant, and anti-inflammatory effects (121). In contrast, 8AGPB incorporates the therapeutic molecule directly into the material architecture. 8-Aminoguanosine serves as a structural component of the supramolecular hydrogel and is released *in situ* as the material degrades, thereby integrating the carrier and bioactive agent within a single therapeutic platform (122). Beyond improving local retention, octopus-inspired nanomicelles enhance adhesion to the periodontal mucosa through phenylboronic acid groups and use fucoidan to target scavenger receptors on macrophages and neutrophils, thereby preferentially delivering cannabidiol to inflammatory cells (123). For these delivery systems, local retention and drug exposure need to be measured alongside tissue outcomes. Increased loading or adhesion alone does not show that the lesion receives an effective, sustained dose.

Beyond enhancing local drug exposure, some biomaterials directly target oxidative stress and metabolic dysregulation in macrophages. Q@MPDA alters energy metabolism in inflammatory macrophages by suppressing glycolysis, enhancing oxidative phosphorylation, and reducing mitochondrial reactive oxygen species (mtROS), thereby attenuating periodontal inflammation and alveolar bone resorption (120). TPP-SAT, in contrast, targets mitochondrial ROS and tissue hypoxia by co-delivering superoxide dismutase (SOD) and catalase (CAT) to mitochondria. By eliminating superoxide anions and hydrogen peroxide while simultaneously generating oxygen, this system alleviates the local microenvironment in which oxidative stress, hypoxia, and macrophage dysfunction mutually reinforce one another (124). Similarly targeting mitochondrial homeostasis, SF-HA@TA-Mn exploits tannic acid and manganese to enhance mitochondrial targeting and ROS scavenging, while modulating the JAK2–STAT1–ASS1 axis, arginine metabolism, and glutamine-associated TCA cycle and purine metabolism. In addition, the SF-HA component provides osteoconductive support for osteogenic differentiation (125). MBG@L-Arg employs a distinct metabolic regulatory strategy. In addition to scavenging ROS, suppressing NF-κB signaling, and activating Nrf2, it releases nitric oxide (NO) and promotes osteogenic differentiation through sGC–cGMP–PKG-associated signaling (126). The cell-based metabolic measurements support mechanistic activity, while the corresponding animal outcomes indicate tissue benefit within each model. These findings make macrophage metabolism a plausible treatment target, but metabolic improvement and bone repair may also reflect effects on different cell populations. Establishing how much of the tissue benefit depends on macrophage metabolic changes would help select which material components are necessary.

To address the periodontal microenvironment, where infection, immune amplification, and bone destruction coexist, multifunctional biomaterials further integrate pathogen control and host-response modulation within a single therapeutic platform. MPB-BA employs mesoporous Prussian blue to load baicalein, enabling photothermal antibacterial activity under near-infrared irradiation. Concurrently, it reduces ROS accumulation and macrophage inflammatory responses by activating Nrf2 and suppressing NF-κB signaling (127). Engineered macrophage membrane-based nanodecoys, in contrast, emphasize pathogen recognition. The retained TLR2 and TLR4, together with membrane-anchored LL-37, enhance bacterial capture and killing. In addition, an internal cascade system comprising L-amino acid oxidase (LAAO) and hollow manganese dioxide increases local oxygen availability while reducing ROS accumulation (128). In contrast, other biomaterials are designed primarily to attenuate sustained amplification of the host inflammatory response rather than to exert direct antibacterial effects. Macrophage membrane-functionalized microspheres sequester TNF-α and IL-6 through membrane-associated receptors and, through the PLGA/β-TCP scaffold, modulate interactions among macrophages, osteoclasts, and bone marrow mesenchymal stem cells (BMSCs), thereby coupling the resolution of inflammation with bone formation (129). Charge-adaptive nanoparticles undergo acid-responsive charge switching to capture pre-existing neutrophil extracellular traps (NETs) while releasing a calcium chelator to suppress further NET formation. This dual action disrupts CXCL3–CXCR2-mediated crosstalk between NETs and macrophages, thereby restoring macrophage mitochondrial function and attenuating inflammation-associated bone resorption (130). In diabetes-associated periodontitis, an ROS- and pH-responsive hydrogel loaded with Ac2–26 and OGP attenuates macrophage pyroptosis through modulation of the ANXA1/NLRP3/caspase-1/GSDMD signaling axis, while OGP supports bone formation in a periodontitis-associated tooth-loss and bone-defect model in diabetic rats (131). For such complex formulations, we consider the contribution of each component an important unresolved question. Comparisons with simpler formulations could test whether the added complexity improves tissue outcomes enough to justify the additional manufacturing and safety requirements (**Table 4**).

The evidence across therapeutic modalities supports a functional, stage-sensitive interpretation of macrophage targeting. Mitophagy restoration, efferocytosis, metabolic remodeling, and altered intercellular communication provide distinct routes to tissue protection (41, 49, 61, 107). However, marker shifts, cytokine reduction, and improved bone measurements are different outcomes and should not be used as substitutes for one another. Studies in human-derived cells or patient tissue do not constitute clinical treatment trials. Likewise, a scaffold or EV preparation that acts on osteoblasts, endothelial cells, or neutrophils as well as macrophages cannot be described as macrophage-specific without appropriate perturbation controls.

We propose that the effect of a macrophage-directed intervention depends on the functional defect present at the time of treatment. Interventions that preserve microbial clearance while reducing damaging inflammatory output may be most relevant during active infection, whereas improving apoptotic-cell clearance and osteogenic support may matter more after control of the microbial stimulus. This proposed sequence remains to be tested. The contrasting consequences of macrophage depletion during inflammatory destruction and subsequent repair provide an experimental rationale for testing timing rather than assuming a universally beneficial polarization direction (58, 60). The contrasting antagomiR-217 outcomes under continued ligation and after ligature removal provide a further, intervention-specific example of this timing dependence (49). To test this hypothesis, the same intervention could be compared with vehicle during continued ligation and after ligature removal, with comparable drug exposure and separate controls for each phase. Bacterial burden, efferocytosis, bone formation, and recurrence should be assessed as separate outcomes. We predict a larger added benefit from restoring efferocytosis during resolution, provided that bacterial control is preserved. A reproducible difference in treatment effect between phases, accompanied by the expected functional changes, would support this prediction. Similar effects in a study capable of detecting a meaningful difference would weaken it; a nonsignificant interaction alone would be inconclusive. The effect of ligature removal must also be separated from that of treatment.

Translation is further limited by uncertain cell specificity, heterogeneous disease models, short observation periods, and incomplete reporting of neutral or adverse outcomes. The nonsignificant gingival transcript responses in the dipyridamole study (71) and the different effects of USP7 silencing and P5091 (75) illustrate why a predominantly positive literature should not be treated as uniform mechanistic confirmation. The immediate priority is reproducible functional target engagement in relevant models, followed by assessment of delivery, safety, and additive benefit over established periodontal care. The representative evidence summarized here does not establish clinical efficacy for these macrophage-directed strategies. Human evidence from adjacent therapeutic approaches provides a more qualified translational perspective. A 127-participant, 28-day phase 1 trial of a lipoxin A4 analog rinse reported preliminary improvement in gingival inflammation, but safety was the primary outcome and the study did not establish macrophage-specific target engagement or periodontal regeneration (142). In a randomized trial involving 30 patients, adding autologous periodontal ligament stem cells to guided tissue regeneration with Bio-Oss did not significantly improve radiographic bone fill over the control treatment through 12 months; both groups improved (143). The trial did not demonstrate added bone-fill benefit from the cells (143); this does not establish equivalence between treatments or exclude benefit under other conditions. We therefore consider benefit over standard care, with prespecified patient-relevant outcomes and adequate follow-up, a more informative translational criterion than improvement from baseline alone. Neither clinical study validates the macrophage-directed formulations reviewed here. An adipose-derived stem-cell EV trial, NCT04270006, is also registered, but its status was unknown and no results were posted when checked on 21 September 2026; the last posted update was 17 February 2020 (144). Registration alone does not demonstrate efficacy, and an unreported outcome should not be classified as treatment failure.

## 6 Conclusions and future perspectives

The periodontal microenvironment regulates macrophage responses through microbial and host-derived signals, hypoxia and oxidative stress, metabolic changes, and epigenetic and post-transcriptional mechanisms. The evidence reviewed identifies heterogeneous macrophage-associated states and experimentally measured changes in selected functions, including inflammatory mediator production, efferocytosis, metabolic activity, and tissue-supportive effects. Selected experimental models also support context-dependent changes in macrophage function, persistent dysfunction, and functional rescue. However, direct evidence demonstrating transitions within the same macrophage population in periodontal lesions remains limited. Macrophage dysregulation in periodontitis is therefore better understood as disruption of specific functional programs and changes in macrophage population composition rather than simply as an altered M1-like to M2-like ratio. Therapeutic studies should identify which macrophage functions are impaired, determine whether those functions can be restored, and test whether such restoration contributes to periodontal benefit. Human associations, experimental mechanisms, and animal tissue protection support distinct conclusions, and none alone establishes patient-level therapeutic efficacy.

Current evidence leaves important questions about periodontal macrophage states and their therapeutic relevance unresolved. Most single-cell studies are cross-sectional. Pseudotime, RNA velocity, and cell–cell communication analyses infer relationships that require experimental testing. A further limitation is the lack of studies linking macrophage identity over time with repeated measurements of function within periodontal lesions. Temporal changes in marker expression or population abundance therefore cannot determine whether existing macrophages have changed their functional state or whether different populations have been recruited, expanded, retained, or lost. Limited sample sizes, inadequate controls, and a lack of functional validation further constrain interpretation (140). Spatial omics has begun to describe local interactions between macrophages and periodontal structural cells, but findings from small cohorts or populations with specific risk factors may not generalize across anatomical sites, disease stages, or populations (70). Experimental models introduce different limitations. Humans and mice differ in myeloid cell composition, inflammatory responses, and intercellular communication, and acute ligature-induced models do not reproduce the chronic, heterogeneous course of human periodontitis (141). Single-LPS stimulation and two-cell co-cultures also omit much of the interaction among multispecies biofilms, hypoxia, metabolic stress, and immune and stromal cells. Several proposed mechanisms of intercellular communication still lack direct causal validation (83). These limitations leave uncertainty about both the mechanisms involved and their value as treatment targets.

The next step is to establish which macrophage functions account for tissue benefit and which can usefully guide treatment. Longitudinal human sampling could relate changes in macrophage population features to measured antimicrobial activity and efferocytosis, while recognizing that repeated tissue samples do not track individual cells. Sequential stimulation experiments should define the initial macrophage condition and measure functional responses after a change in environmental cues. In periodontal models, lineage or fate tracking combined with time-resolved functional assays could help distinguish functional changes within existing macrophages from recruitment or replacement of different populations. Cell-specific perturbation and rescue could then test whether the identified functional changes are required for tissue repair. We also propose that baseline functional impairment may predict benefit from a matched intervention better than the proportion of M2-like cells. For a candidate with adequate safety evidence and feasible functional assays, both measures could be collected before treatment and compared in a randomized study against standard care. The comparison should assess whether either measure identifies a difference in treatment benefit, rather than merely a difference in prognosis, and should be validated independently. If functional measurements offer no additional predictive value, the proposal should be revised. These studies would help determine whether measuring macrophage function can improve treatment decisions; they do not yet provide a rule for selecting patients.

## Glossary

3′UTR: 3′ untranslated region
α-KG: α-Ketoglutarate
APA: Alternative polyadenylation
ATP: Adenosine triphosphate
BAFF: B-cell activating factor
BMDMs: Bone marrow-derived macrophages
BMSCs: Bone marrow mesenchymal stem cells
CAT: Catalase
CCL2: C-C motif chemokine ligand 2
CCR2: C-C motif chemokine receptor 2
CR3: Complement receptor 3
DALYs: Disability-adjusted life years
DCIR: Dendritic cell immunoreceptor
DHFR: Dihydrofolate reductase
DMOG: Dimethyloxalylglycine
GTR: Guided tissue regeneration
H3K27ac: Histone H3 lysine 27 acetylation
H3K56ac: Histone H3 lysine 56 acetylation
HIF-1α: Hypoxia-inducible factor-1α
IFN-γ: Interferon-γ
IFN-I: Type I interferons
IL: Interleukin
iMSCs: Induced pluripotent stem cell-derived mesenchymal stem cells
iNOS: Inducible nitric oxide synthase
KAT6A: Lysine acetyltransferase 6A
LAAO: L-amino acid oxidase
LPS: Lipopolysaccharide
Ltx: Leukotoxin
m^6^A: N^6^-methyladenosine
miRNAs: MicroRNAs
MMPs: Matrix metalloproteinases
mtDNA: Mitochondrial DNA
mtROS: Mitochondrial reactive oxygen species
MyD88: Myeloid differentiation primary response 88
NETs: Neutrophil extracellular traps
NELL1: Neural epidermal growth factor-like 1
NF-κB: Nuclear factor-κB
NO: Nitric oxide
OGP: Osteogenic growth peptide
OMVs: Outer membrane vesicles
PDLSCs: Periodontal ligament stem cells
PI3K: Phosphoinositide 3-kinase
PI3Kγ: Phosphoinositide 3-kinase gamma
RANK: Receptor activator of nuclear factor-κB
RANKL: Receptor activator of nuclear factor-κB ligand
ROS: Reactive oxygen species
RvD2: Resolvin D2
SAA1: Serum amyloid A1
SAM: S-adenosylmethionine
SCFA: Short-chain fatty acid
SIRT6: Sirtuin 6
SOD: Superoxide dismutase
SOCE: Store-operated calcium entry
TCA: Tricarboxylic acid
Th1: T helper 1
TIM4: T-cell immunoglobulin and mucin domain-containing protein 4
TLR2: Toll-like receptor 2
TUFM: Mitochondrial translation elongation factor Tu
UCHL1: Ubiquitin C-terminal hydrolase L1
